# The global burden of female breast cancer attributable to high fasting plasma glucose: A comprehensive analysis of the GBD database from 1990 to 2023

**DOI:** 10.1371/journal.pone.0359268

**Published:** 2026-10-21

**Authors:** Huadi Shi, Zhi Song, Yaoyao Chen, Siyuan Liu, Yuling Song

**Affiliations:** 1 Affiliated Hospital of Guangdong Medical University, Zhanjiang, Guangdong Province, China; 2 Third Division General Hospital, Xinjiang Production and Construction Corps, Tumushuke, Xinjiang, China; 3 Zhanjiang Central Hospital, Guangdong Medical University, Zhanjiang, Guangdong Province, China; 4 The Second Affiliated Hospital of Guangdong Medical University, Zhanjiang, Guangdong Province, China; National Center for Chronic and Noncommunicable Disease Control and Prevention, Chinese Center for Disease Control and Prevention, CHINA

## Abstract

**Background:**

High fasting plasma glucose (HFPG) is an established risk factor for breast cancer (BC). However, accurate and up-to-date estimates of the global burden of breast cancer attributable to high fasting plasma glucose (BC-HFPG) are scarce. This study aims to provide a comprehensive analysis of the burden of BC-HFPG using data from the Global Burden of Disease (GBD) database from 1990 to2023.

**Methods:**

We performed a comprehensive descriptive analysis of the burden of female BC-HFPG based on the GBD 2023 database. The core metrics analyzed were the absolute number of death and disability-adjusted life years (DALY). To ensure comparability across populations with differing age structures, we also analyzed the age-standardized mortality rate (ASMR) and the age-standardized DALY rate (ASDR). The average annual percent change (AAPC) was used to further comprehensively describe the burden of disease and its changing trends. In addition to the retrospective analysis from 1990 to 2023, we used the Bayesian age-period-cohort (BAPC) model to generate predicted data on the burden of female BC-HFPG from 2024 to 2050.

**Results:**

Female BC-HFPG accounted for 48,307 deaths and 1,406,186 DALYs globally in 2023. From 1990 to 2023, the global ASMR and ASDR of female BC-HFPG showed overall upward trends, with an average annual percentage change (AAPC) of 0.28% (95% CI: 0.18% to 0.38%) for ASMR and 0.44% (95% CI: 0.37% to 0.51%) for ASDR; the two rates increased from 0.92 and 26.22 per 100,000 in 1990 to 1.00 and 30.17 per 100,000 in 2023, respectively. The burden varied substantially across the 21 GBD regions and 204 countries and territories in 2023. In 2023, SDI was only weakly correlated with ASMR (Spearman ρ = 0.20, P = 0.004) and not significantly correlated with ASDR, and the burden peaked in low-middle SDI quintile countries. The BAPC model projected that the ASMR and ASDR of female BC-HFPG would continue to rise from 2024 to 2050.

**Conclusion:**

The global burden of female BC-HFPG increased from 1990 to 2023 and is projected to continue rising through 2050, disproportionately affecting low- and low-middle SDI regions. These findings suggest that glycemic management and metabolic risk-factor control warrant consideration within comprehensive breast cancer prevention frameworks. However, because this is an ecological, population-level analysis, whether glucose lowering interventions can reduce breast cancer incidence remains to be established in prospective interventional studies. Our results may help prioritize regions and populations for future etiological and intervention research.

## 1. Introduction

Breast cancer remains one of the most common malignant tumors in women worldwide. In 2022, there were approximately 2.3 million new cases and 660 000 deaths worldwide, ranking second for cancer-related deaths among women [1]. In recent years, many new therapeutic agents have emerged in the field of breast cancer, including new target inhibitors, antibody drug conjugates, and immune checkpoint inhibitors, which have significantly improved patient survival [2]. However, once distant metastasis occurs, breast cancer is generally considered incurable, underscoring the critical importance of primary prevention [3]. Early identification of high-risk groups of breast cancer and minimizing exposure to known breast cancer risk factors, such as environmental factors, lifestyle behaviors, dietary habits, and metabolic factors, are essential for the prevention of breast cancer [4]. Therefore, a detailed understanding of the local cancer burden and its associated risk factors is essential for formulating effective national cancer control policies and targeted interventions.

Cancer metabolism is a hallmark of cancer progression [5]. Chronic high blood sugar may be associated with cancer formation through the DNA damage caused by enhanced oxidative stress resulting from mitochondrial glucose oxidation [6]. A body of evidence supports an association between diabetes and a 23% increased risk of breast cancer [7]. Although that study defined exposure as clinically diagnosed diabetes—a narrower construct than the HFPG definition adopted in GBD analyses, in which fasting plasma glucose above the theoretical minimum risk level of 4.8–5.4 mmol/L additionally encompasses pre-diabetic hyperglycemia-it provides supportive evidence that glucose dysmetabolism increases breast cancer risk. Directly relevant to HFPG, a retrospective cohort study based on data from the Korean National Health Insurance Service (NHIS) revealed a positive correlation between FPG levels of 5.6–6.9 mmol/L and breast cancer incidence compared to individuals with FPG < 5.6mmol/L [8]. Collectively, these studies based on related but distinct exposure definitions suggest a positive association between HFPG levels and the risk of breast cancer, indicating that hyperglycemia may be an independent risk factor for its development.

Currently, large-scale global studies on the burden of female breast cancer attributable to HFPG are limited, and updated analyses are lacking. Recently, the Global Burden of Disease (GBD) database has been updated with data for 2022 and 2023. Herein, we present the findings of the global burden of female breast cancer attributable to high fasting plasma glucose in 2023. This study describes the deaths, DALYs, AAPC from 1990 to 2023 at the global level, across 21 regions, 204 countries, and various Socio-demographic Index (SDI) categories. The aim of this research is to inform and guide cancer control efforts through evidence-based policy-making, resource allocation, and health system planning.

## 2. Methods

### 2.1. Data acquisition

We obtained data from the Global Burden of Disease (GBD) database for the period 1990–2023. This database encompasses estimates for 371 diseases and injuries and 88 risk factors across 204 countries and regions. The global burden of femal BC-HFPG risk factors was analyzed. This is detailed in the global burden of disease report (https://www.healthdata.org/) [9]. All the data obtained in this study were from public databases, so they did not involve ethical issues required in the design of medical research.

In the GBD comparative risk assessment framework, high fasting plasma glucose (HFPG) is defined as fasting plasma glucose exceeding the Theoretical Minimum-Risk Exposure Level (TMREL)-the population-level exposure that would minimize disease risk. According to the GBD 2023 risk factor analysis, the TMREL for fasting plasma glucose corresponds to approximately 4.8–5.4 mmol/L(86.4–97.2 mg/dL). It should be emphasized that HFPG as defined here represents a continuous exposure above the TMREL and is not equivalent to a clinical diagnosis of diabetes mellitus; it captures the full spectrum of elevated fasting glucose, including prediabetic ranges. Accordingly, the burden estimates reported in this study represent the fraction of female breast cancer burden attributable to HFPG exposure under the counterfactual of the TMREL, as modeled by GBD using pooled relative risks from prospective cohort studies.

The analysis was restricted to female breast cancer because the GBD comparative risk assessment includes high fasting plasma glucose as an established risk factor for female breast cancer only; to date, no risk–outcome pair between HFPG and male breast cancer has been modeled in the GBD framework, owing to the rarity of male breast cancer and the limited evidence base for quantitative risk estimation.

### 2.2. Ethics approval and consent to participate

The GBD 2023 project is an open-access database in which all data are de-identified and publicly available. The University of Washington’s Institutional Review Board reviewed and approved an informed consent waiver (https://www.healthdata.org/research-analysis/gbd). No additional ethical approval was required for this secondary analysis of anonymized, publicly available data.

### 2.3. Statistical analysis

Trends in deaths and DALYs of BC-HFPG from 1990 to 2023 were assessed by calculating the average annual percent change (AAPC) and using Joinpoint regression [10]. The model starts with the minimum number of joinpoints (0) and tests whether adding more points (up to a maximum of 5) is statistically significant (using the Bonferroni correction). The resulting model provides the Annual Percentage Change (APC) for each segment and the Average Annual Percentage Change (AAPC) over the entire period. Log-linear regression model was used to calculate the AAPC and its 95% confidence interval (CI) and analyze the trend. The test level α = 0.05, and the analysis were conducted by joinpoint software (version 5.4; https://surveillance.cancer.gov/joinpoint/) [11]. All data collation and computational analyses were performed with the use of open source R software (version 4.5.1; https://www.r-project.org/). To formally quantify the ecological association between socio-demographic development and disease burden, Pearson (linear) and Spearman (rank-based) correlation coefficients were calculated between the 2023 SDI and the ASMR/ASDR across the 204 countries and territories, with 95% confidence intervals (CIs) derived from 1,000 bootstrap resamples. Locally estimated scatterplot smoothing (LOESS) curves were fitted to characterize the shape of the SDI-burden relationship. To account for regional dependence among countries, a sensitivity analysis was repeated at the level of the 21 GBD regions. Differences in ASMR and ASDR across the five SDI quintiles (low, low-middle, middle, high-middle, and high) were compared using the Kruskal-Wallis test. These correlation analyses were performed with Python (version 3.13; SciPy and statsmodels packages), and all such results were interpreted as ecological associations rather than individual-level causal relationships. We constructed a Bayesian age-period-cohort (BAPC) model to generate predicted data on the burden of female BC-HFPG from 2024 to 2050 [12]. To assess the out-of-sample predictive performance of the BAPC model, we conducted a temporal validation using data from 1990 to 2015 for model fitting and the held-out 2016–2023 period for validation (retroactive prediction mode). Age-specific counts were modelled with a Bayesian age–period–cohort framework using integrated nested Laplace approximations (INLA), with random walks of second order (RW2) priors for the age, period, and cohort effects and a Poisson likelihood. Age-standardized rates were computed using the mean 1990–2023 global female age structure as the internal standard. Predictive accuracy was quantified by the mean absolute percentage error (MAPE), the root mean squared error (RMSE), and the coverage of the 95% prediction intervals. All analyses were performed in R 4.3.2 using the BAPC and INLA packages. All values presented for 2024–2050 are model-based projections generated by the BAPC model and do not represent observed estimates; official GBD empirical estimates are currently available through 2023 only. The data were fitted using the R software packages “INLA” and “BAPC.” The projected population data for the period from 2024 to 2050 were obtained from “the GBD Population Forecasts 2017–2100.” When the p-value is less than 0.05, it is considered to have statistical significance.

## 3. Results

### 3.1. Global burden of disease

Globally, female BC-HFPG accounted for 19,328 deaths (95% UI: 12,236–31,629) in 1990, with an ASMR of 0.92 per 100,000 population; by 2023, deaths had increased to 48,307 (95% UI: 32,467–71,815), with an ASMR of 1.00 per 100,000 population. Over the same period, DALYs attributable to female BC-HFPG increased from 569,715 (95% UI: 344,450–955,897) in 1990, with an ASDR of 26.22 per 100,000 population, to 1,406,186 in 2023 (95% UI:921,533-2,130,980), with an ASDR of 30.17 per 100,000 population (Table 1). The joinpoint analysis indicated three phases in the temporal trends of the global ASMR (AAPC: 0.28%, 95% CI: 0.18–0.38%) and ASDR (AAPC: 0.44%, 95% CI: 0.37–0.51%): a gradual increase during 1990–1993, a sustained decline during 1994–2013, and a rapid upward trend during 2014–2023. Overall, these AAPCs indicated upward trends over the entire 1990–2023 period (Fig 1). Notably, although the absolute number of BC-HFPG deaths increased by 150.0% (from 19,328 in 1990–48,307 in 2023), the ASMR rose by only 8.7% (from 0.92 to 1.00 per 100,000). This divergence indicates that the growth in the absolute burden was largely driven by population growth and demographic aging, whereas the modest rise in age-standardized rates reflects an increase in individual-level metabolic risk beyond demographic change.

**Table 1:** The number and age-standardized rates of deaths and DALYs of female BC-HFPG in global and 21 regions, as well as AAPC from 1990 to 2023.

Location	Deaths			Dalys		
	ASMR_1990	ASMR_2023	AAPC	ASDR_1990	ASDR_2023	AAPC
Global	0.92 (0.58-1.5)	1 (0.67-1.5)	0.28 (0.18 - 0.38)	26.22 (15.97-43.78)	30.17 (19.67-45.88)	0.44 (0.37 - 0.51)
Southeast Asia	0.47 (0.3-0.8)	0.87 (0.58-1.36)	1.88 (1.74 - 2.02)	14.55 (9.01-25.68)	28.09 (18.52-45.38)	1.98 (1.84 - 2.12)
Central Asia	0.79 (0.48-1.32)	0.71 (0.45-1.09)	−0.15 (−0.69 - 0.4)	23.65 (14.29-40.96)	20.91 (13.07-32.48)	−0.31 (−0.88 - 0.27)
Oceania	0.99 (0.53-1.81)	1.65 (1.01-2.74)	1.6 (1.41 - 1.79)	29.46 (15.48-53.74)	49.92 (29.9-86.09)	1.63 (1.42 - 1.85)
East Asia	0.71 (0.41-1.15)	0.47 (0.32-0.68)	−1.31 (−1.77 - −0.84)	24.75 (13.72-39.26)	14.85 (9.93-22.19)	−1.55 (−1.78 - −1.31)
Central Europe	1.17 (0.61-2.21)	1.49 (0.86-2.24)	0.73 (0.66 - 0.81)	31.85 (15.67-62.65)	38.2 (20.59-61.61)	0.55 (0.47 - 0.62)
Eastern Europe	0.68 (0.36-1.32)	0.89 (0.49-1.48)	0.81 (0.28 - 1.35)	20.61 (10.75-41.3)	25.16 (13.75-43.4)	0.57 (0.14 - 1)
High-income Asia Pacific	0.47 (0.28-0.72)	0.7 (0.45-1.02)	1.2 (1.04 - 1.37)	15.41 (8.78-24.73)	20.71 (12.87-31.22)	0.91 (0.62 - 1.19)
Australasia	1.03 (0.62-1.82)	1.02 (0.62-1.7)	−0.07 (−0.58 - 0.43)	27.06 (15.63-48.51)	27.8 (16.39-48.28)	0.06 (−0.36 - 0.48)
Western Europe	1.56 (1.02-2.47)	1.25 (0.91-1.72)	−0.68 (−0.82 - −0.54)	42.13 (26.23-69.09)	32.95 (22.67-44.8)	−0.75 (−0.9 - −0.6)
Southern Latin America	1.38 (0.9-2.19)	1.26 (0.89-1.77)	−0.2 (−0.44 - 0.05)	36.73 (22.83-59.83)	34.41 (23.64-49.61)	−0.1 (−0.36 - 0.17)
Caribbean	0.85 (0.55-1.33)	1.22 (0.85-1.74)	1.05 (0.82 - 1.28)	24.5 (15.48-38.98)	34.65 (23.9-49.35)	1.03 (0.81 - 1.25)
Andean Latin America	0.33 (0.19-0.64)	0.82 (0.5-1.3)	2.91 (2.53 - 3.29)	9.59 (5.19-18.35)	24.82 (14.97-39.85)	2.96 (2.47 - 3.45)
High-income North America	1.47 (0.93-2.48)	1.18 (0.75-1.83)	−0.66 (−0.82 - −0.5)	42.04 (25.83-72)	33.85 (21.29-52.46)	−0.66 (−0.83 - −0.49)
Tropical Latin America	1.45 (0.85-1.96)	1.46 (0.86-2.03)	0.06 (−0.21 - 0.33)	43.4 (25.07-59.21)	43.16 (24.34-62.06)	−0.04 (−0.19 - 0.12)
Central Latin America	0.78 (0.53-1.05)	1.07 (0.81-1.4)	0.96 (0.76 - 1.17)	23.01 (15.12-31.6)	32.13 (24.28-41.58)	1 (0.78 - 1.22)
South Asia	0.46 (0.26-0.84)	1.05 (0.59-1.82)	2.49 (2.11 - 2.87)	14.17 (7.7-26.12)	31.76 (17.62-55.62)	2.45 (1.96 - 2.94)
North Africa and Middle East	0.49 (0.29-0.9)	1.21 (0.85-1.72)	2.81 (2.64 - 2.97)	14.52 (8.52-26.9)	37.64 (25.93-54.22)	2.94 (2.77 - 3.11)
Eastern Sub-Saharan Africa	0.23 (0.13-0.42)	0.59 (0.34-1.09)	2.96 (2.83 - 3.09)	7.31 (4.05-13.93)	19.42 (11.02-36.31)	3.03 (2.87 - 3.18)
Western Sub-Saharan Africa	0.67 (0.31-1.43)	1.78 (0.86-3.48)	3.03 (2.82 - 3.23)	20.56 (9.53-44.19)	56.46 (26.96-112.04)	3.13 (2.96 - 3.31)
Central Sub-Saharan Africa	1.11 (0.57-2.06)	2.77 (1.54-4.96)	2.82 (2.58 - 3.07)	39.82 (20-75.39)	104.93 (57.37-193.37)	2.97 (2.72 - 3.23)
Southern Sub-Saharan Africa	0.63 (0.39-1.15)	1.53 (0.96-2.2)	2.77 (2.4 - 3.15)	18.02 (10.66-34.68)	45.31 (27.78-67.7)	2.9 (2.33 - 3.47)

**Fig 1:**
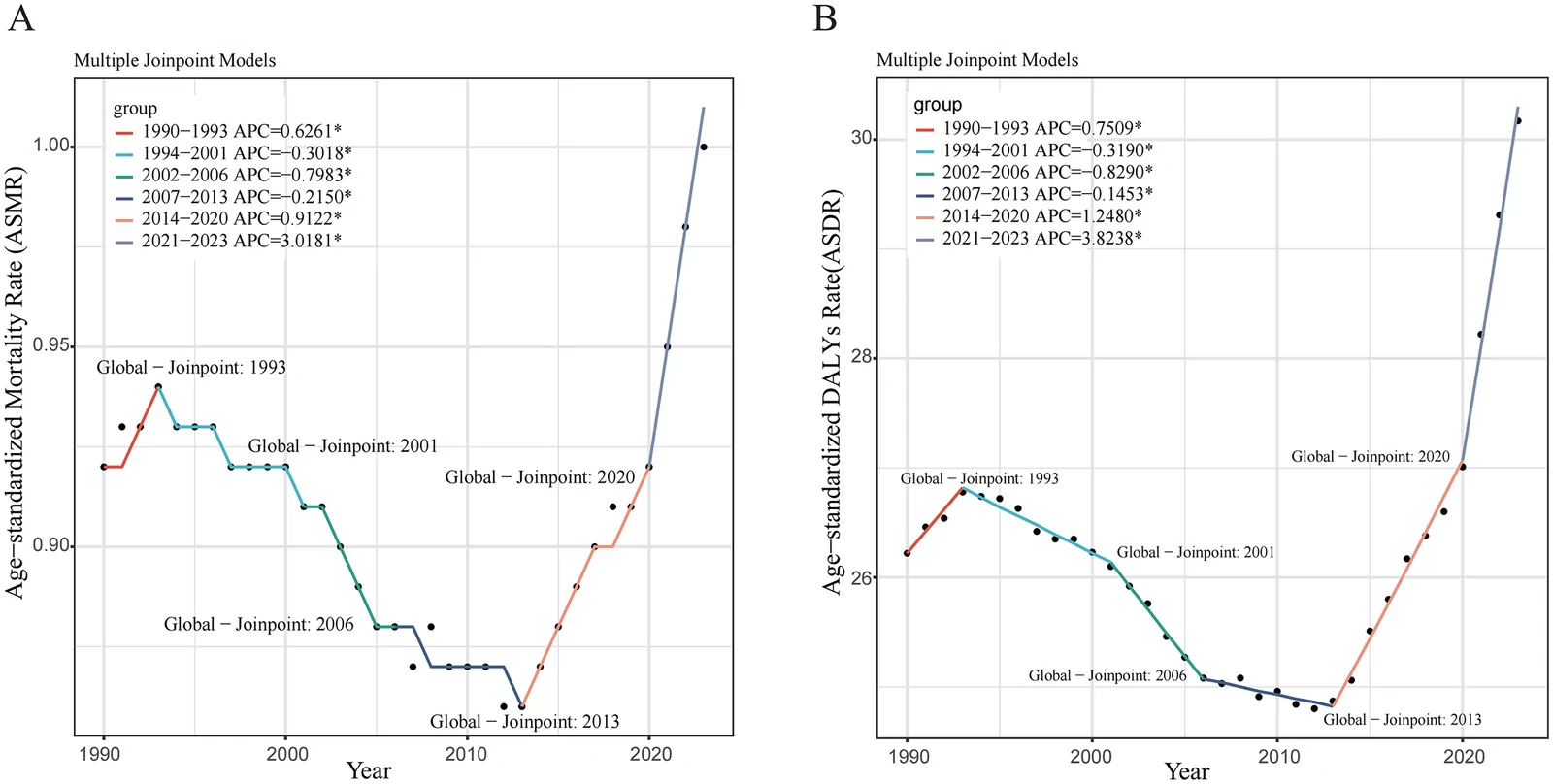
Joinpoint regression analysis results in global. (A) Deaths. (B) DALYs. *P < 0.05.

### 3.2. Regional burden of disease

Among the 21 regions, Central Sub-Saharan Africa has the highest ASMR(2.77 cases per 100,000 people) and ASDR(104.93cases per 100,000 people) of female BC-HFPG, which is approximately 3 times that of the global in 2023. Notably, North Africa and Middle East (deaths:AAPC = 2.81; dalys:AAPC = 2.94), Central Sub-Saharan Africa (deaths:AAPC = 2.82; dalys:AAPC = 2.97), Eastern Sub-Saharan Africa (deaths:AAPC = 2.96; dalys:AAPC = 3.03), and Western Sub-Saharan Africa (deaths:AAPC = 3.03; dalys:AAPC = 3.13) have seen continuous increases in ASMR, and ASDR from 1990 to 2023. In contrast, East Asia (deaths:AAPC = −1.31; dalys:AAPC = −1.55), Western Europe (deaths:AAPC = −0.68; dalys:AAPC = −0.75) and High-income North America (deaths:AAPC = −0.66; dalys:AAPC = −0.66) have decreased in ASMR and ASDR over the past 34 years (Table 1).

### 3.3. 204 countries’ burden of disease

There are significant differences in the disease burden of breast cancer **attributable to** high fasting plasma glucose (HFPG) among 204 countries worldwide in 2023. The age-standardized mortality rate (ASMR) was the highest in Marshall, with 6.77 cases per 100,000 people (95% UI: 4.21–10.17), while it was the lowest in Mozambique, at 0.1 case per 100,000 people (95% UI: 0.05–0.21). Conversely, Nauru had the highest age-standardized DALY rate (ASDR), reaching 200.67 cases per 100,000 people (95% UI: 112.87–335.25), while in Mozambique it was the lowest, at only 2.65 case per 100,000 people (95% UI: 1.31–5.26). Trend analysis shows that the ASMR in 153 countries across the world significantly increased from 1990 to 2023, with Arab Republic of Egypt experiencing the most significant increase (AAPC:5.34%; 95%CI:4.63–6.06). In addition, 23 countries experienced a decline, among which Kingdom of Spain saw the most significant drop (AAPC:-1.5; 95%CI:-1.75 - −1.25). In terms of DALYs, 152 countries witnessed significant increases, with Arab Republic of Egypt experiencing the greatest growth(AAPC:4.98; 95%CI:4.42–5.55); while 38 countries saw a decline, and People’s Republic of China had the most significant decrease (AAPC:-1.73; 95%CI:-1.98 - −1.48)(Supplementary S1 Table).

### 3.4. Associations with the SDI

Over the past three decades, the SDI in most of the 21 regions increased alongside ASMR and ASDR (e.g., North Africa and Middle East, Western Sub-Saharan Africa, South Asia, and Eastern Sub-Saharan Africa), whereas in High-income North America, East Asia, and Western Europe, ASMR and ASDR declined despite continuously rising SDI (Fig 2). To formally quantify this ecological association, Pearson and Spearman correlation coefficients were calculated between the 2023 SDI and ASMR/ASDR across the 204 countries and territories. SDI showed only a weak positive correlation with ASMR (Pearson r = 0.13, 95% CI: 0.02 to 0.24, P = 0.073; Spearman ρ = 0.20, 95% CI: 0.06 to 0.32, P = 0.004) and no significant correlation with ASDR (Pearson r = 0.03, 95% CI: −0.08 to 0.15; Spearman ρ = 0.09, 95%CI: −0.04 to 0.24, P = 0.196). LOESS curves revealed a non-linear, inverted-U-shaped relationship, with both ASMR and ASDR peaking at an SDI of approximately 0.75 (Fig 3). In a sensitivity analysis at the level of the 21 GBD regions, the correlations attenuated and reversed direction (ASMR: r = −0.37, P = 0.101; ASDR: r = −0.46, P = 0.036) (Fig 4), indicating that the association was scale-dependent and should be interpreted with caution against the ecological fallacy. Using GBD’s official SDI quintile classification, both ASMR and ASDR in 2023 were highest in the low-middle SDI quintile (ASMR 1.22 per 100,000, 95% UI: 0.70–2.12; ASDR 37.7 per 100,000, 95%UI: 21.7–67.5), and the inter-quintile differences were statistically significant (Kruskal-Wallis H = 29.9 for ASMR and 25.7 for ASDR, both P < 0.001). The low-SDI quintile had a 1.18-fold higher ASDR than the high-SDI quintile (32.6 vs 27.6 per 100,000), whereas ASMR was comparable between the two quintiles (0.98 vs 0.98 per 100,000). Collectively, the HFPG-attributable breast cancer burden was non-linearly distributed across the development spectrum, peaking in low-middle SDI countries rather than being uniformly concentrated in less-developed areas.

**Fig 2:**
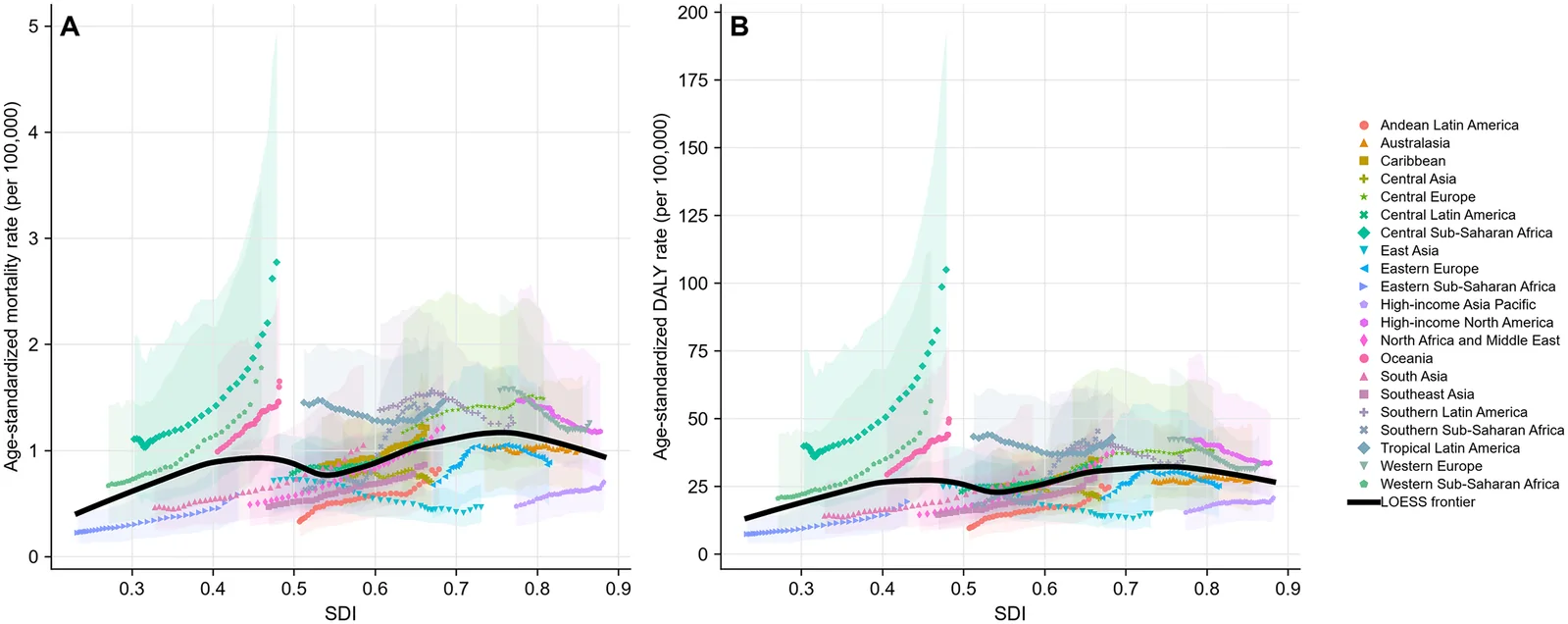
Frontier analysis of the age-standardized mortality rate (ASMR, A) and the age-standardized DALY rate (ASDR, B) of female breast cancer attributable to high fasting plasma glucose versus the Socio-demographic Index (SDI) across the 21 GBD regions, 1990–2023. Colored trajectories show regional observed GBD estimates from 1990 to 2023. Error bars (plotted at 5-year intervals for readability) denote the 95% uncertainty intervals of the GBD estimates. The solid black line is a locally estimated scatterplot smoothing (LOESS) frontier fitted to all region–year observations.

**Fig 3:**
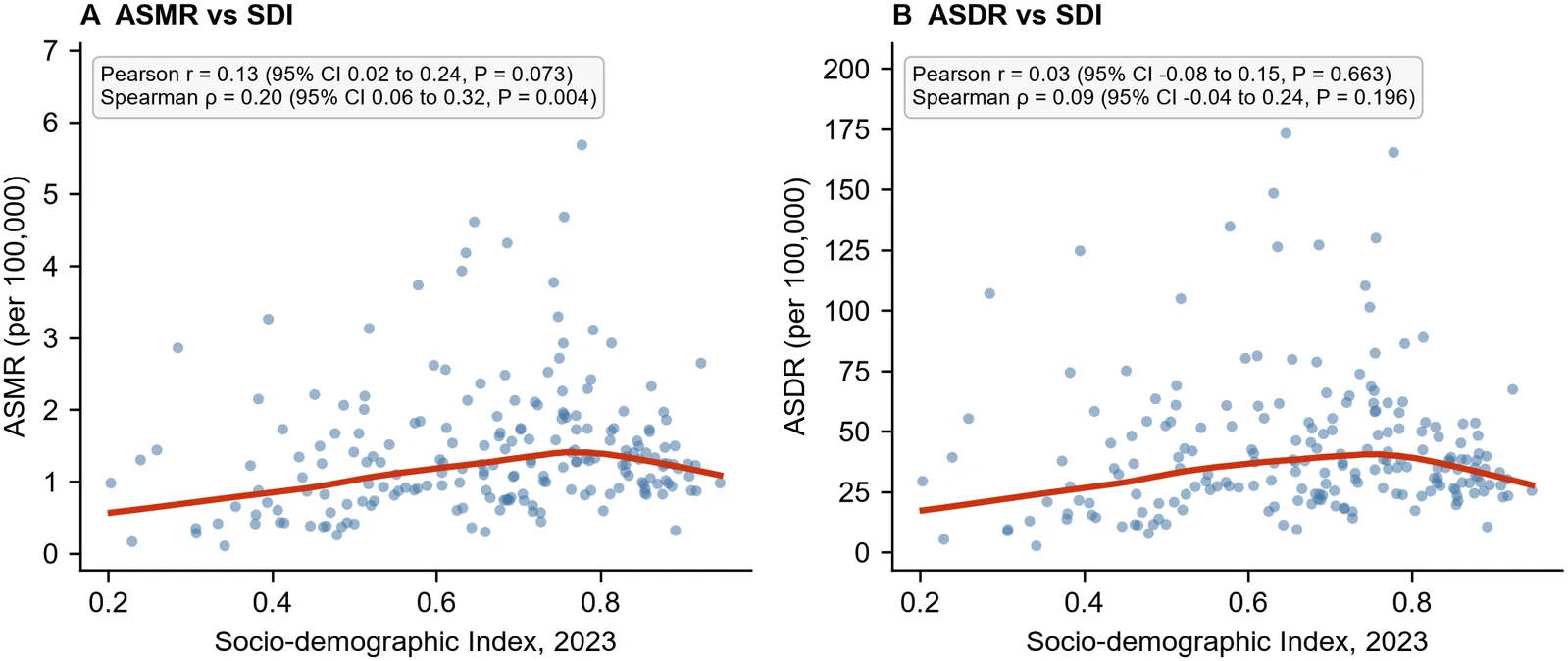
Associations between the Socio-demographic Index (SDI) and the age-standardized mortality rate (ASMR; A) and age-standardized disability-adjusted life year rate (ASDR; B) of breast cancer attributable to high fasting plasma glucose across 204 countries and territories, females, 2023. Each point represents one country. Solid curves show locally estimated scatterplot smoothing (LOESS) fits with 95% confidence bands (shaded areas), revealing an inverted-U-shaped relationship with the burden peaking at an SDI of approximately 0.75. Pearson (r) and Spearman (ρ) correlation coefficients with 95% confidence intervals derived from 1,000 bootstrap resamples are shown in each panel.

**Fig 4:**
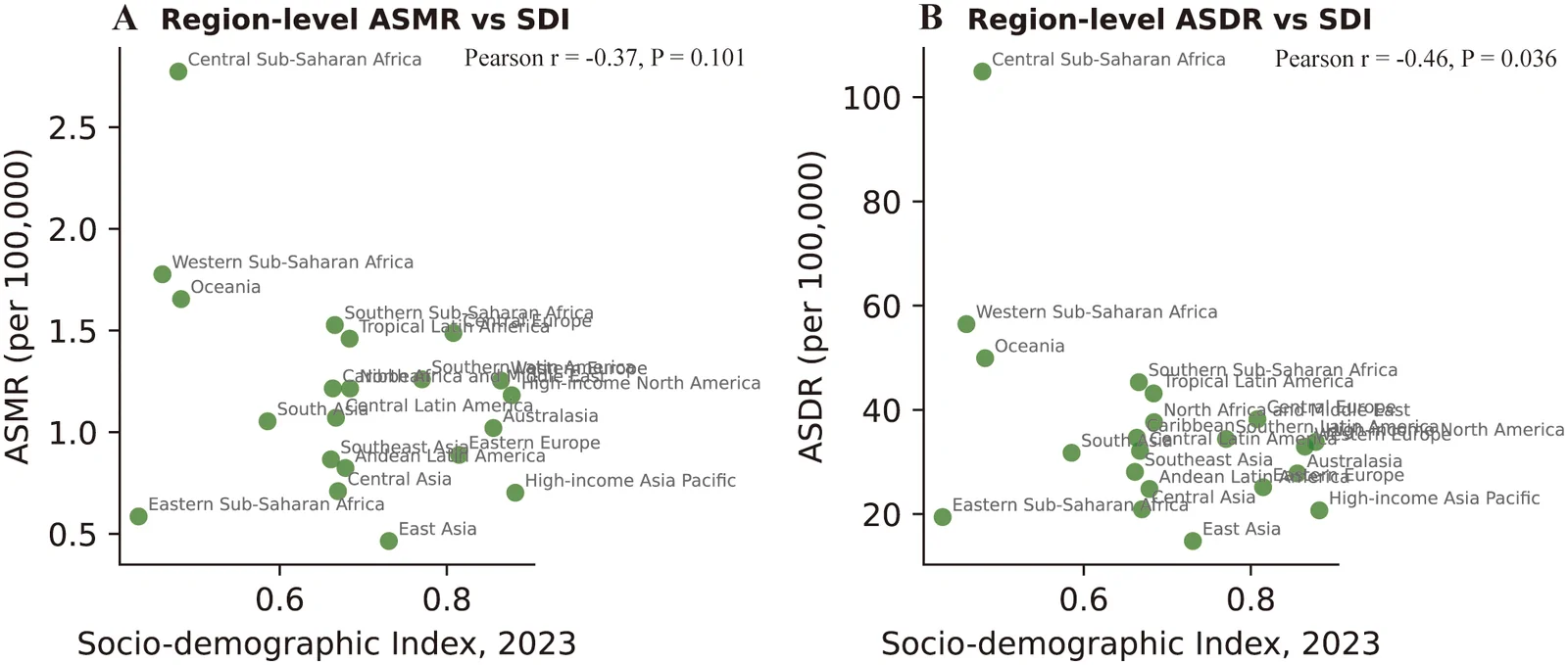
Sensitivity analysis of the SDI–burden association at the level of the 21 Global Burden of Disease (GBD) regions, females, 2023: SDI versus ASMR (A) and ASDR (B). Each point represents one GBD region; dashed lines show linear regression fits. Compared with the country-level analysis, the correlations attenuated and reversed direction, indicating that the SDI–burden association is scale-dependent.

### 3.5. Global burden predictions for BC-HFPG from 2024 to 2050

The Bayesian age-period-cohort model (BAPC) was used to predict the burden of BC-HFPG from 2024 to 2050. The results of the prediction showed that the ASMR and ASDR of female BC-HFPG were expected to continue to rise steadily until 2050. By 2050, it was predicted that the ASMR would be 2.7 cases per 100,000 people, and the ASDR would be 95.9 cases per 100,000 people (Fig 5).

**Fig 5:**
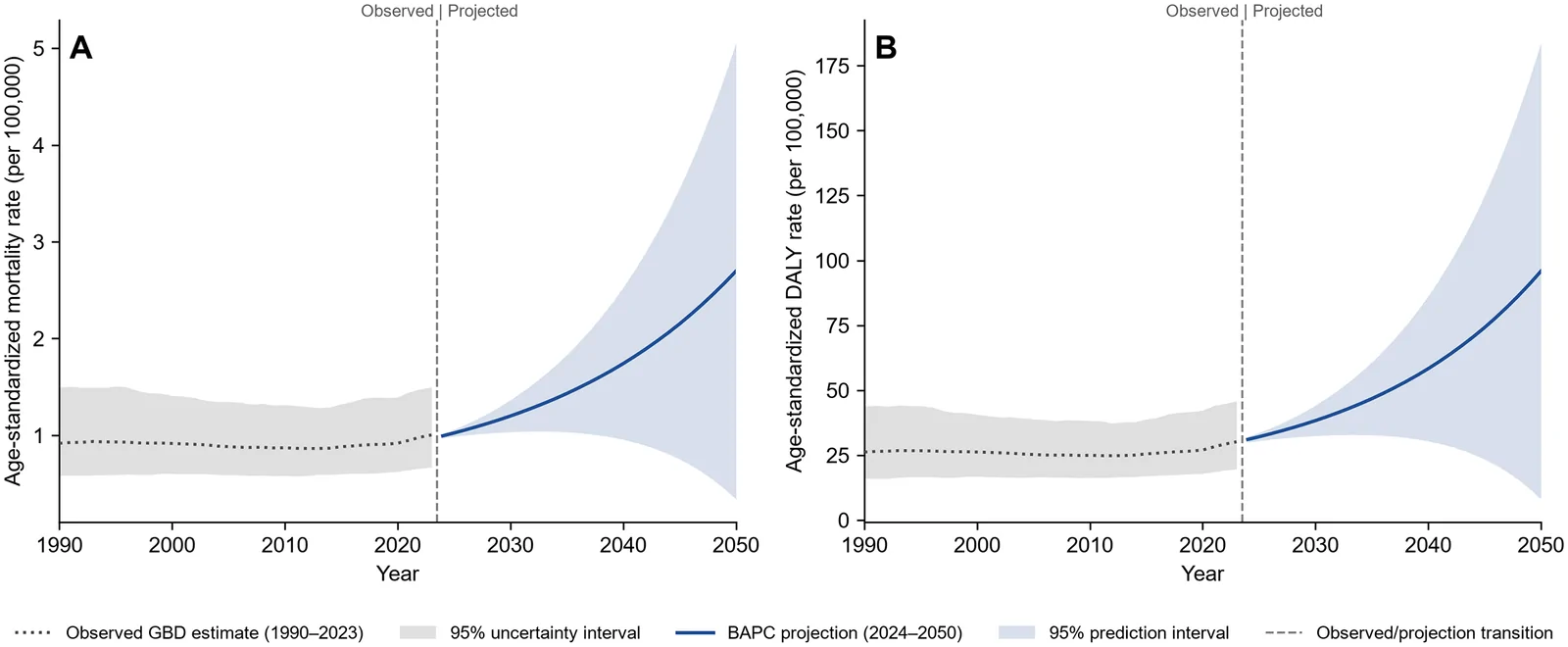
Predictions for the age-standardized mortality rate (ASMR, A) and the age-standardized DALY rate (ASDR, B) of global female breast cancer attributable to high fasting plasma glucose (BC-HFPG), 1990-2050. Dotted lines and grey shaded bands denote observed GBD 2023 estimates with 95% uncertainty intervals (1990-2023). Solid lines and blue shaded bands denote model-based Bayesian age-period-cohort (BAPC) projections with 95% prediction intervals (2024–2050). The vertical dashed line marks the transition from observed estimates to model projections. Values for 2024-2050 are model-based projections, not observed estimates.

In the out of sample validation, the BAPC model fitted on 1990–2015 data reproduced the observed 2016–2023 age-standardized rates with high accuracy: the MAPE was 1.88% for ASMR (RMSE = 0.051 per 100,000) and 2.06% for ASDR (RMSE = 1.485 per 100,000), and all eight observed values fell within the corresponding 95% prediction intervals (coverage 100%). Prediction errors increased monotonically with the forecast horizon, from below 1.3% within the first five years to approximately 6% by 2023, and the model systematically under-predicted the accelerated burden observed in 2021–2023(Fig 6). These findings support the reliability of short-term projections while underscoring that long-range extrapolations, including those to 2050, should be interpreted with caution.

**Fig 6:**
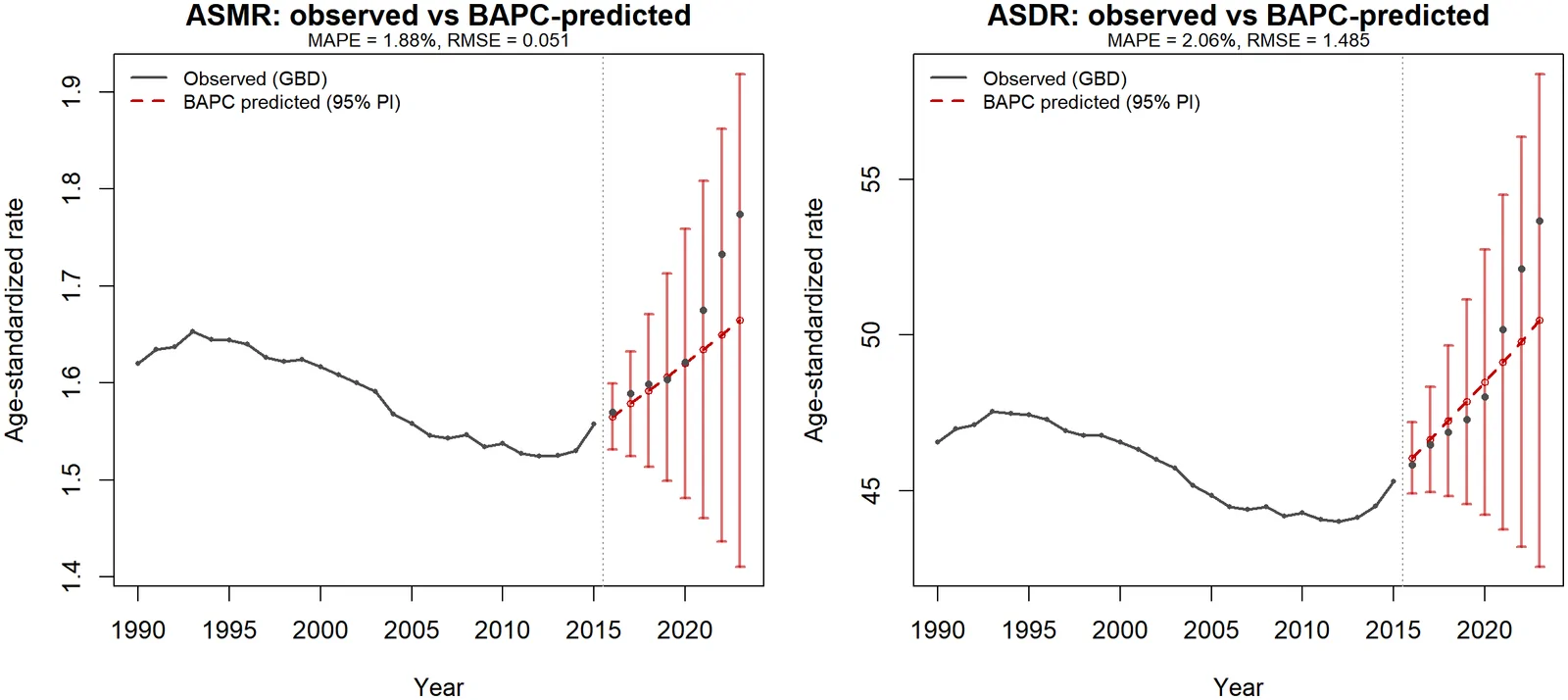
Out of sample validation of the Bayesian age period cohort (BAPC) model for ASMR and ASDR of global female BC-HFPG from 1990 to 2023. The model was fitted to 1990-2015 data (left of the dotted vertical line) and used to predict the held out 2016-2023 period. Solid lines denote GBD-observed rates; red dashed lines with error bars denote BAPC retrospective predictions with 95% prediction intervals.

## 4. Discussion

This research presents the first comprehensive assessment of the global burden of female BC-HFPG based on the GBD data from 1990 to 2023. The results reveal a sustained upward trend in BC-HFPG deaths and DALYs, with significant regional heterogeneity. The BAPC model projected that the ASMR and ASDR of female BC-HFPG would continue to rise from 2024 to 2050. These findings provide a critical evidence base for formulating targeted prevention and control strategies.

Epidemiological studies have shown that hyperglycemia can affect the progression of tumors in various types of cancer, including breast cancer [13,14]. Previous studies have shown that HFPG is closely related to the occurrence, development, recurrence and metastasis of breast cancer [15,16]. The mechanism by which HFPG promotes the development and progression of breast cancer is very complex and involves multiple metabolic pathways. The series of changes **attributable to** hyperglycemia eventually lead to dysregulation of cellular signaling pathways (including Wnt/β-catenin, Notch, NF-κB, PI3K/Akt/mTOR and MAPK signaling pathways, etc.), which subsequently trigger oxidative stress, endoplasmic reticulum stress, and the failure of the unfolded protein response (UPR) related to glucose toxicity. At the same time, it is accompanied by abnormal autophagy, cell and DNA damage, and inhibition of breast cell apoptosis, thereby initiating or promoting the occurrence of cancer in breast cells [17]. These multiple mechanisms collectively explain the strong association between HFPG and breast cancer. It should be noted, however, that much of this mechanistic evidence derives from models of diabetic hyperglycemia; while supportive, the exposure quantified in the present study (GBD-defined HFPG) is broader and also captures elevated fasting glucose below the diabetic threshold.

This study shows that the ASMR and ASDR of BC-HFPG in Central Sub-Saharan Africa are approximately three times the global average, with a rapid increase, while East Asia, Western Europe, and high-income North America show a declining trend. These disparities are closely related to the socioeconomic and healthcare levels of the respective regions. It should be noted that this SDI-burden association is ecological and non-linear: SDI alone explained only a small fraction of the cross-country variation (Pearson r = 0.13 for ASMR), and the correlation attenuated and reversed direction when countries were aggregated into the 21 GBD regions (r =−0.46 for ASDR). Accordingly, these correlations should not be interpreted as individual-level causal relationships. The inverted-U-shaped curve-peaking in low-middle SDI countries-plausibly reflects a transition phase in which diabetes prevalence and obesity are rising rapidly while breast cancer survival gains and screening/treatment access lag behind those in high-SDI countries. The high burden in low- and low-middle SDI regions is primarily attributable to: poor accessibility to healthcare resources; for instance, some parts of Africa have fewer than 5 radiotherapy machines per million people [18], coupled with low coverage of blood glucose screening and diabetes management gaps that also imply limited detection and control of elevated fasting glucose among women without diagnosed diabetes [19]; delayed breast cancer diagnosis, where the proportion of late-stage (III/IV) breast cancer diagnoses in low-income countries is as high as 50–80%, compared to only 10–20% in high-income countries [20]; and inadequate management of metabolic diseases [21]. In contrast, high SDI regions have effectively alleviated the medical burden by implementing a series of measures, including establishing screening systems for blood sugar and breast cancer. For instance, in Europe and the United States, glucose monitoring has been included in the routine physical examinations for high-risk breast cancer patients [22,23]. Particularly noteworthy is the important role of unbalanced nutritional transition. In low- and middle-SDI countries undergoing rapid urbanization, the prevalence of high-sugar and high-fat diets is incompatible with the environment lacking in health education and sports facilities, leading to a sharp increase in the incidence of metabolic diseases [24]. On the contrary, the traditional dietary patterns in East Asia are typically rich in dietary fiber and have a low glycemic load, which offer protection for metabolic health [25].

Several caveats apply to the interpretation of temporal trends. First, oncogenesis following metabolic exposure involves a substantial lag period; the burden observed in any given year partly reflects HFPG exposure from years to decades earlier, meaning that recent changes in diabetes screening and management will only become visible in BC-HFPG trends after a delay. Second, the aggregate GBD estimates do not permit formal decomposition of the observed trends into components attributable to changing HFPG prevalence, improvements in breast cancer survival, intensification of diagnosis and screening, and population aging. The divergent trajectories of absolute counts versus age-standardized rates (a 150% increase in deaths versus a 8.7% increase in ASMR) suggest that demographic change is the dominant driver of the absolute burden, while changes in survival and diagnostic intensity likely also contribute to the age-standardized trends. Future studies employing formal decomposition methods (e.g., age-period-cohort decomposition) are needed to disentangle these components.

Based on these research results, we suggest that for high-burden regions (such as sub-Saharan Central Africa), a comprehensive primary healthcare system should be established as a priority. Blood glucose testing should be incorporated into the existing breast cancer screening programs, and mobile medical technologies should be utilized to address the accessibility of services in remote areas. Furthermore, in order to enhance women’s understanding of the correlation between metabolic health and the risk of breast cancer, community health education programs should be actively carried out. At the same time, the frequency of breast ultrasound examinations for women with abnormal blood sugar levels should be increased. Countries like Kenya, which have a low burden but limited resources, should enhance the collection of basic data and establish cancer registration systems to avoid underestimating the burden. Furthermore, by exploring the risk stratification model assisted by artificial intelligence, integrating multiple dimensions of parameters such as blood sugar changes, BMI index, and family medical history, innovative prevention and control strategies are adopted to accurately identify high-risk individuals [26]. At the same time, efforts should be made to promote research on metabolic reprogramming in order to develop precise prevention strategies for BC-HFPG.

This study explores the disease burden of BC-HFPG in different countries and regions, providing information for targeted public health strategies. However, this study has several limitations: The quality of reports varies among different countries. In some countries, there might be cases of data underreporting, which could affect the accuracy of the GBD data; The definition of HFPG does not include more stable indicators like HbA1c. Moreover, because GBD defines HFPG as fasting plasma glucose above the theoretical minimum risk level rather than a clinical diagnosis of diabetes, our attributable burden estimates are not directly comparable with studies of diabetes-attributable breast cancer burden. Furthermore, the analysis could not examine the specific association between breast cancer molecular subtypes and HFPG. Therefore, the conclusions of this study should be interpreted with caution, especially in regions with a lower disease burden. Looking to the future, it is necessary to integrate additional metabolic risk factors—such as obesity, physical inactivity, and dietary patterns—alongside HFPG, and explore their interactions with breast cancer molecular subtypes. Such efforts will be crucial for developing personalized prevention strategies. Finally, our projections to 2050 assume that historical exposure and mortality trends continue unchanged. The rapid global uptake of novel metabolic therapies—most notably GLP-1 receptor agonists—together with expanding diabetes screening and management programmes could substantially alter future HFPG exposure trajectories; if these interventions translate into sustained population-level reductions in fasting glucose, the projected rise in BC-HFPG burden may be overestimated. Our forecasts should therefore be interpreted as a reference scenario conditional on the continuation of current trends rather than an inevitable future.

## 5. Conclusion

The burden of BC-HFPG has increased globally and disproportionately affects low- and low-middle SDI regions. These findings suggest that glycemic management and metabolic risk-factor control warrant consideration within comprehensive breast cancer prevention frameworks; however, as this is an ecological, population-level analysis, whether glucose-lowering interventions can reduce breast cancer incidence remains to be established in prospective interventional studies. Our results may help prioritize regions and populations for future etiological and intervention research.

## Supporting information

S1 Table The number and age-standardized rates of deaths and DALYs of female BC-HFPG in 204 countries, as well as AAPC from 1990 to 2023. (DOCX)
