# Supplementary material for: The global burden of female breast cancer attributable to high fasting plasma glucose: A comprehensive analysis of the GBD database from 1990 to 2023
Source: PLoS One. 2026 Oct 21;21(10):e0359268. doi: 10.1371/journal.pone.0359268 (PMC13686481; doi:10.1371/journal.pone.0359268)
Supplement: Supplementary file 1 [file pone.0359268.s005.pdf]

General Comments

This manuscript utilizes the Global Burden of Disease (GBD) 2023 dataset to describe the mortality and disability-adjusted life years (DALYs) of female breast cancer attributable to high fasting plasma glucose (HFPG). The authors also attempted to respond to the reviewers' queries very well. Unfortunately, the current submission still suffers from significant methodological opacity, conceptual overreach, and a lack of statistical validation for its projections. The manuscript, in its present form, functions as a descriptive data update but fails to meet the rigorous standards for causal inference and predictive modeling despite attempts to upscale it after revision.

Major Compelling Points (Methodology and Design)

1. Conceptual and causal overreach

a) Serious concern: The manuscript repeatedly claims breast cancer is "caused by" HFPG. GBD comparative-risk assessments calculate *attributable* burden based on population exposure and pre-existing risk-outcome relationships; they do not establish causality within a new analysis. The authors must replace "caused by" with "attributable to" throughout the text, including the title.

b) Prevention recommendations: It is clear that the conclusion that these findings highlight an "urgency" for blood-glucose control as a breast cancer prevention strategy exceeds the evidence. This study is an ecological observation, not a clinical trial. It does not demonstrate that glycemic intervention reduces cancer incidence. I think the list to perfect here is framing as hypotheses for further investigation.

2. Methodological Scope

a) The authors treat HFPG interchangeably with diabetes. They must explicitly define the GBD exposure metric, specifically the Theoretical Minimum-Risk Exposure Level (TMREL). Without knowing the counterfactual (e.g., 4.8–5.4 mmol/L), the burden estimates lack biological context.

b) The analysis is restricted to "female" breast cancer without justification. While rare, male breast cancer is a recognized entity. The authors must state if this restriction is due to a lack of GBD risk-outcome pairs for males or a specific study design choice.

c) "Systematic Analysis"? This is a secondary analysis of a public database. Labeling it a "systematic analysis" is misleading as it does not follow PRISMA or similar systematic review protocols.

3. I have observed the authors attributed the rise in ASMR/ASDR solely to HFPG. However, they fail to account for the lag-time effect (the biological period between glucose exposure and oncogenesis). Furthermore, they must decompose the trend: how much is driven by changing HFPG prevalence versus changes in breast cancer survival, diagnostic intensity, or population aging?

Also, the authors conflate absolute numbers (48,307 deaths) with age-standardized rates. An increase in absolute burden is often a function of population growth and demographic aging rather than an increase in individual-level metabolic risk. These must be distinguished in the discussion.

4. The claim that SDI and BC-HFPG burden are linked is currently descriptive and prone to the Ecological Fallacy. Visual upward movement of two variables over 30 years does not establish an epidemiological link. A formal regression analysis or correlation coefficient (accounting for regional dependence) is required.

The term "developing regions" is not a formal GBD classification. The authors must use SDI quintiles (Low, Low-Middle, etc.) and provide quantitative proof that these areas are disproportionately affected compared to High-SDI regions.

5. Deficiencies in the BAPC forecasting model are grossly noted.

a) The Bayesian age-period-cohort (BAPC) model is a "black box" in this manuscript. The authors must provide out-of-sample validation (e.g., training the model on 1990–2015 data to see if it accurately predicts the observed 2016–2023 GBD estimates).
b) The projection begins in 2024, yet the current evaluation date is August 2026. The authors must explain why they are "predicting" years for which empirical data may already be accessible and clarify that 2024–2050 are model-based projections, not observed estimates.
c) The 2050 linear projection fails to account for the current global shift in metabolic management (e.g., the rapid uptake of GLP-1 receptor agonists). The authors must discuss how revolutionary changes in global glucose management might render their 26-year linear forecast obsolete.

Minor and Technical Issues

1. The Results state a trend analysis from **1992–2023**, yet the reported values and Table 1 refer to **1990–2023**. This inconsistency must be resolved to ensure the integrity of the AAPC estimates.
2. Average Annual Percent Changes (AAPC) are reported as 0.28% and 0.44%. The authors must provide 95% Confidence Intervals (CIs) in the text (not just tables) and state whether these changes are statistically distinguishable from zero.
3. Figures 2 and 3 must include uncertainty intervals to prevent an appearance of false precision in the long-term forecasts.
