# Supplementary material for: The global burden of female breast cancer attributable to high fasting plasma glucose: A comprehensive analysis of the GBD database from 1990 to 2023
Source: PLoS One. 2026 Oct 21;21(10):e0359268. doi: 10.1371/journal.pone.0359268 (PMC13686481; doi:10.1371/journal.pone.0359268)
Supplement: Supplementary file 2 [file pone.0359268.s001.docx]

**Table S1. The number and age-standardized rates of deaths and DALYs of female BC-HFPG in 204 countries, as well**

**as AAPC from 1990 to 2023.**

| **Location** | **Deaths** | | | **Dalys** | | |
| --- | --- | --- | --- | --- | --- | --- |
|  | **ASR_1990** | **ASR_2023** | **AAPC** | **ASR_1990** | **ASR_2023** | **AAPC** |
| People's Republic of China | 0.72 (0.41-1.17) | 0.44 (0.31-0.66) | -1.48 (-1.99 - -0.98) | 25.1 (13.94-39.97) | 14.16 (9.47-21.14) | -1.73 (-1.98 - -1.48) |
| Democratic People's Republic of Korea | 0.43 (0.17-0.95) | 0.9 (0.41-1.89) | 2.26 (2.12 - 2.4) | 12.9 (4.92-28.72) | 27.34 (12.31-61.17) | 2.28 (2.14 - 2.43) |
| Lao People's Democratic Republic | 0.4 (0.2-0.77) | 1.27 (0.69-2.44) | 3.6 (3.46 - 3.74) | 11.9 (6.02-22.87) | 39.33 (21.36-76.22) | 3.72 (3.58 - 3.88) |
| Kingdom of Cambodia | 0.21 (0.1-0.4) | 0.41 (0.22-0.75) | 2.01 (1.8 - 2.22) | 6.36 (2.81-12.69) | 11.6 (6.45-21.26) | 1.83 (1.56 - 2.1) |
| Taiwan (Province of China) | 0.67 (0.43-1.07) | 1.25 (0.78-1.72) | 1.83 (1.61 - 2.05) | 21 (13.01-34.41) | 40.81 (24.58-57.23) | 1.92 (1.73 - 2.1) |
| Republic of Indonesia | 0.4 (0.21-0.79) | 0.77 (0.44-1.43) | 2.03 (1.84 - 2.22) | 12.66 (6.93-24.95) | 26.26 (14.73-48.24) | 2.25 (2.05 - 2.45) |
| Republic of the Union of Myanmar | 0.75 (0.4-1.4) | 1.27 (0.74-2.25) | 1.57 (1.26 - 1.87) | 24.34 (12.95-46.55) | 41.91 (24.42-74.82) | 1.55 (1.24 - 1.85) |
| Malaysia | 1.13 (0.63-1.79) | 1.92 (1.08-2.72) | 1.52 (1.3 - 1.74) | 32.66 (17.99-52.56) | 61.8 (33.84-90.31) | 1.89 (1.68 - 2.11) |
| Democratic Socialist Republic of Sri Lanka | 0.46 (0.28-0.77) | 1.08 (0.69-1.73) | 2.65 (2.13 - 3.18) | 13.4 (7.61-22.57) | 32.8 (20.38-54.83) | 2.9 (2.39 - 3.4) |
| Republic of the Philippines | 0.7 (0.43-1.17) | 1.23 (0.81-2.01) | 1.82 (1.57 - 2.08) | 19.82 (12.36-33.08) | 37.88 (24.86-62.64) | 2.02 (1.88 - 2.15) |
| Kingdom of Thailand | 0.54 (0.31-0.9) | 0.75 (0.48-1.2) | 1 (0.29 - 1.71) | 16.55 (9.45-27.33) | 24.04 (15.12-39.86) | 1.15 (0.52 - 1.78) |
| Republic of Maldives | 0.45 (0.2-0.92) | 0.88 (0.48-1.59) | 2.01 (1.86 - 2.16) | 13.25 (6-27.62) | 21.35 (11.5-38.04) | 1.43 (1.25 - 1.61) |
| Socialist Republic of Viet Nam | 0.17 (0.08-0.34) | 0.36 (0.2-0.68) | 2.28 (2.14 - 2.41) | 5.09 (2.53-9.86) | 11.26 (6.2-21.88) | 2.42 (2.28 - 2.57) |
| Democratic Republic of Timor-Leste | 0.21 (0.09-0.44) | 0.37 (0.19-0.75) | 1.65 (1.26 - 2.03) | 5.77 (2.45-12.31) | 11.53 (5.79-24.05) | 2.06 (1.74 - 2.38) |
| Republic of Kiribati | 0.73 (0.4-1.23) | 1.51 (0.92-2.34) | 2.24 (1.97 - 2.5) | 24.76 (13.13-42.33) | 51.52 (32.04-80.11) | 2.27 (1.93 - 2.61) |
| Republic of Fiji | 2.1 (1.1-3.67) | 4.32 (3.12-6.21) | 2.21 (2.06 - 2.35) | 64.2 (32.8-111.95) | 127.06 (93.35-180.44) | 2.07 (1.91 - 2.22) |
| Independent State of Papua New Guinea | 0.64 (0.3-1.4) | 1.06 (0.53-2.05) | 1.57 (1.21 - 1.93) | 19.8 (9.01-42.67) | 34.72 (17.07-69.52) | 1.75 (1.35 - 2.14) |
| Republic of the Marshall Islands | 3.12 (1.99-5.26) | 6.77 (4.21-10.16) | 2.38 (2.26 - 2.49) | 85.27 (54.54-138.57) | 184.27 (114.62-273.87) | 2.38 (2.25 - 2.51) |
| Independent State of Samoa | 0.69 (0.36-1.26) | 1.31 (0.78-2.14) | 1.93 (1.79 - 2.08) | 23.2 (11.72-42.51) | 42.49 (25.81-68.28) | 1.85 (1.66 - 2.04) |
| Federated States of Micronesia | 1.91 (1.08-3.22) | 2.62 (1.72-3.88) | 0.99 (0.8 - 1.17) | 57.27 (32.87-96.94) | 80.22 (51.52-118.9) | 1.03 (0.85 - 1.22) |
| Kingdom of Tonga | 1.97 (1.08-3.24) | 4.18 (2.58-6.29) | 2.31 (2.03 - 2.59) | 61.79 (33.48-100.18) | 126.29 (79.16-188.11) | 2.15 (1.96 - 2.33) |
| Republic of Azerbaijan | 0.75 (0.47-1.36) | 0.83 (0.48-1.44) | 0.23 (-0.55 - 1.01) | 24 (14.52-44.37) | 25.41 (14.57-45.13) | 0.11 (-0.59 - 0.83) |
| Solomon Islands | 0.66 (0.33-1.3) | 1.49 (0.78-2.85) | 2.5 (1.9 - 3.11) | 18.97 (9.48-38.8) | 48.09 (24.82-93.45) | 2.86 (2.25 - 3.48) |
| Republic of Vanuatu | 0.7 (0.34-1.47) | 1.67 (0.85-3.01) | 2.45 (1.88 - 3.03) | 20.65 (9.79-44.37) | 54.18 (27.56-100.26) | 2.74 (2.14 - 3.35) |
| Republic of Armenia | 0.98 (0.61-1.6) | 0.67 (0.45-1.01) | -1.11 (-1.88 - -0.34) | 29.17 (17.96-48.9) | 18 (11.93-28.43) | -1.42 (-2.12 - -0.72) |
| Georgia | 1 (0.58-1.62) | 1.2 (0.77-1.95) | 0.54 (-0.04 - 1.13) | 31.38 (17.7-52.77) | 34.21 (21.52-56.46) | 0.18 (-0.58 - 0.94) |
| Kyrgyz Republic | 0.68 (0.4-1.2) | 0.6 (0.37-1.03) | -0.39 (-1.45 - 0.68) | 20.22 (11.48-36.64) | 16.95 (10.09-30.29) | -0.53 (-1.5 - 0.46) |
| Mongolia | 0.18 (0.1-0.36) | 0.3 (0.18-0.55) | 1.45 (1.11 - 1.78) | 5.59 (3.01-11.76) | 9.48 (5.59-17.57) | 1.47 (1.11 - 1.84) |
| Republic of Kazakhstan | 0.84 (0.51-1.41) | 0.57 (0.33-0.94) | -1.18 (-1.81 - -0.54) | 24.27 (14.56-41.66) | 16.76 (9.51-28.81) | -1.12 (-1.69 - -0.55) |
| Turkmenistan | 0.62 (0.35-1.17) | 0.74 (0.44-1.25) | 0.63 (-0.2 - 1.47) | 19.16 (10.45-37.14) | 24.16 (13.92-42.59) | 0.8 (-0.09 - 1.69) |
| Republic of Tajikistan | 0.87 (0.47-1.54) | 1.1 (0.63-1.75) | 0.8 (0.25 - 1.35) | 27.47 (14.41-49.77) | 32.19 (17.82-52.16) | 0.54 (-0.01 - 1.11) |
| Republic of Uzbekistan | 0.68 (0.42-1.14) | 0.63 (0.42-0.97) | -0.23 (-0.91 - 0.46) | 19.81 (11.65-33.59) | 18.82 (12.28-28.26) | -0.12 (-0.87 - 0.64) |
| Republic of Albania | 0.45 (0.21-0.91) | 0.73 (0.35-1.28) | 1.58 (1.34 - 1.82) | 11.36 (5.14-23.09) | 19.15 (8.66-34.47) | 1.7 (1.41 - 2) |
| Republic of Bulgaria | 1.12 (0.63-1.94) | 1.28 (0.76-2.04) | 0.4 (-0.28 - 1.09) | 32.4 (17.58-59.38) | 34.55 (19.66-55.98) | 0.17 (-0.5 - 0.85) |
| Hungary | 1.31 (0.77-2.36) | 1.32 (0.8-2.17) | 0.01 (-0.14 - 0.16) | 35.9 (20.62-67.24) | 35.31 (20.35-58.59) | -0.09 (-0.23 - 0.06) |
| Republic of Croatia | 1.27 (0.64-2.43) | 1.32 (0.72-2.09) | 0.18 (-0.15 - 0.51) | 31.82 (15.17-63.43) | 35.05 (18.22-57.81) | 0.35 (0.06 - 0.64) |
| Bosnia and Herzegovina | 0.73 (0.34-1.45) | 1.59 (0.76-2.55) | 2.42 (2.25 - 2.6) | 20.19 (8.93-42.12) | 40.69 (19.67-66.28) | 2.19 (2.02 - 2.36) |
| North Macedonia | 1.05 (0.49-2.29) | 1.89 (0.92-3.05) | 1.89 (1.47 - 2.32) | 28.4 (12.77-63.34) | 48.48 (23.22-81.19) | 1.7 (1.28 - 2.12) |
| Czech Republic | 1.56 (0.78-2.94) | 1.24 (0.7-1.93) | -0.73 (-1.06 - -0.41) | 40.2 (19.23-78.28) | 30.71 (16.41-50.78) | -0.8 (-1.17 - -0.43) |
| Republic of Poland | 1.22 (0.71-2.21) | 1.49 (0.93-2.15) | 0.59 (0.34 - 0.85) | 33.78 (18.31-63.47) | 37.56 (22.23-56.71) | 0.3 (0.12 - 0.48) |
| Montenegro | 1.37 (0.68-2.78) | 2.42 (1.21-4.14) | 1.74 (1.57 - 1.91) | 35.68 (17.13-74.3) | 62.25 (31.58-105.42) | 1.75 (1.51 - 2) |
| Slovak Republic | 1.4 (0.73-2.4) | 1.71 (0.93-2.46) | 0.64 (0.42 - 0.87) | 38.13 (18.83-69.13) | 42.37 (22.98-61.91) | 0.36 (0.14 - 0.57) |
| Romania | 0.79 (0.34-1.7) | 1.44 (0.69-2.4) | 1.85 (1.66 - 2.03) | 23.3 (9.62-51.59) | 38.07 (17.42-66.13) | 1.5 (1.31 - 1.69) |
| Republic of Serbia | 1.26 (0.55-2.76) | 2.29 (1.15-3.55) | 1.77 (1.62 - 1.93) | 33.43 (14.62-76.19) | 57.77 (27.69-90.54) | 1.65 (1.5 - 1.79) |
| Republic of Slovenia | 1.51 (0.74-2.79) | 1.56 (0.85-2.35) | 0.09 (-0.48 - 0.66) | 39.75 (18.1-75.17) | 38.18 (19.58-58.05) | -0.14 (-0.56 - 0.28) |
| Republic of Belarus | 0.38 (0.23-0.64) | 0.59 (0.37-0.96) | 1.15 (0.03 - 2.29) | 11.65 (6.84-19.97) | 17.21 (10.31-28.98) | 1.03 (-0.08 - 2.15) |
| Republic of Latvia | 1.03 (0.52-2.04) | 0.99 (0.52-1.81) | -0.05 (-0.63 - 0.54) | 29.39 (14.03-59.57) | 26.5 (13.57-49.99) | -0.27 (-0.92 - 0.38) |
| Russian Federation | 0.66 (0.35-1.29) | 0.91 (0.52-1.48) | 1 (0.7 - 1.31) | 20.29 (10.54-40.53) | 25.27 (13.94-42.72) | 0.66 (0.22 - 1.1) |
| Republic of Lithuania | 0.74 (0.35-1.59) | 0.82 (0.39-1.56) | 0.23 (-0.18 - 0.65) | 20.92 (9.54-45.33) | 21.21 (9.88-42.08) | -0.05 (-0.45 - 0.34) |
| Republic of Estonia | 1.07 (0.58-2.13) | 0.89 (0.51-1.62) | -0.57 (-0.7 - -0.44) | 30.72 (16.27-62.62) | 23.39 (13.06-43.17) | -1.07 (-1.55 - -0.59) |
| Republic of Moldova | 0.88 (0.51-1.59) | 0.99 (0.59-1.64) | 0.5 (-0.68 - 1.69) | 26.98 (14.92-50.18) | 28.82 (16.6-48.65) | 0.31 (-0.85 - 1.48) |
| Ukraine | 0.73 (0.36-1.44) | 0.89 (0.44-1.58) | 0.59 (-0.19 - 1.38) | 21.85 (10.5-44.53) | 26.6 (13.29-49.3) | 0.59 (-0.13 - 1.32) |
| Japan | 0.5 (0.29-0.76) | 0.82 (0.49-1.22) | 1.52 (1.33 - 1.72) | 16.27 (9-25.74) | 24.38 (14.5-38.24) | 1.24 (0.89 - 1.58) |
| Republic of Korea | 0.29 (0.18-0.5) | 0.32 (0.19-0.51) | 0.27 (0.05 - 0.49) | 9.74 (5.88-17.37) | 10.53 (6.17-17.47) | 0.18 (-0.08 - 0.45) |
| Brunei Darussalam | 0.92 (0.52-1.81) | 1.35 (0.71-2.28) | 1.17 (1.06 - 1.27) | 29.26 (16.34-58.3) | 47.73 (25.08-80.96) | 1.5 (1.38 - 1.63) |
| Australia | 0.96 (0.58-1.63) | 0.96 (0.59-1.6) | 0.03 (-0.38 - 0.44) | 24.69 (14.72-42.88) | 25.86 (15.44-44.93) | 0.16 (-0.22 - 0.54) |
| Republic of Singapore | 1.08 (0.62-1.9) | 0.97 (0.56-1.52) | -0.34 (-0.84 - 0.15) | 34.49 (19.31-61.76) | 28.67 (15.6-46.26) | -0.54 (-0.98 - -0.1) |
| New Zealand | 1.42 (0.72-2.84) | 1.33 (0.75-2.27) | -0.22 (-0.77 - 0.34) | 38.93 (19.07-79.75) | 38.32 (20.84-66.04) | -0.07 (-0.57 - 0.45) |
| Kingdom of Belgium | 1.52 (0.78-3.07) | 1.25 (0.65-2.26) | -0.57 (-0.82 - -0.31) | 41.19 (20.12-87.22) | 31.92 (16.05-59.61) | -0.76 (-1.02 - -0.5) |
| Principality of Andorra | 0.75 (0.4-1.7) | 1.02 (0.54-2.12) | 0.93 (0.84 - 1.01) | 20.78 (10.73-48.65) | 31.2 (15.92-67.63) | 1.25 (1.18 - 1.32) |
| Republic of Finland | 1.19 (0.77-1.9) | 1.49 (0.94-2.11) | 0.63 (0.35 - 0.92) | 31.49 (20.08-51.93) | 39.73 (24.82-58.6) | 0.75 (0.43 - 1.07) |
| Republic of Cyprus | 0.9 (0.42-2.07) | 1.4 (0.69-2.82) | 1.35 (1.07 - 1.64) | 22.62 (10.59-52.22) | 34.38 (16.22-69.59) | 1.3 (0.96 - 1.64) |
| Republic of Austria | 0.98 (0.58-1.8) | 0.84 (0.48-1.55) | -0.48 (-0.74 - -0.22) | 25.19 (14.08-48.33) | 19.65 (10.8-37.08) | -0.76 (-1.05 - -0.46) |
| Federal Republic of Germany | 1.58 (1.03-2.46) | 1.47 (1.07-1.96) | -0.22 (-0.39 - -0.06) | 41.88 (26.69-66.45) | 38 (25.23-54.61) | -0.32 (-0.52 - -0.12) |
| Kingdom of Denmark | 1.91 (1.08-3.56) | 1.22 (0.64-2.24) | -1.28 (-1.81 - -0.75) | 50.47 (28.14-95.22) | 30.29 (15-57.6) | -1.52 (-2.01 - -1.03) |
| Ireland | 1.45 (0.86-2.67) | 1.24 (0.71-2.19) | -0.45 (-0.92 - 0.01) | 40.04 (22.35-75.72) | 33.06 (17.95-60.15) | -0.63 (-1.12 - -0.14) |
| Hellenic Republic | 1.19 (0.8-1.73) | 1.43 (0.84-2.32) | 0.54 (0.13 - 0.95) | 31.48 (20.57-46.86) | 34.94 (19.73-58.31) | 0.3 (-0.03 - 0.62) |
| French Republic | 1.05 (0.67-1.63) | 1.04 (0.58-1.85) | -0.01 (-0.32 - 0.31) | 29.06 (17.89-47.04) | 28.88 (15.39-52.42) | -0.02 (-0.48 - 0.45) |
| Republic of Iceland | 1.37 (0.7-2.62) | 1.24 (0.63-2.2) | -0.29 (-1.14 - 0.57) | 38.58 (18.23-77.36) | 34.51 (16.76-62.73) | -0.52 (-1.46 - 0.43) |
| State of Israel | 1.57 (0.81-3.02) | 1.4 (0.78-2.39) | -0.37 (-0.64 - -0.1) | 41.79 (20.18-83.04) | 35.06 (18.58-62.6) | -0.59 (-0.79 - -0.39) |
| Grand Duchy of Luxembourg | 1.52 (0.87-2.63) | 1.18 (0.7-2.04) | -0.92 (-1.12 - -0.72) | 39.88 (21.79-69.2) | 27.84 (16.32-47.47) | -1.19 (-1.31 - -1.07) |
| Republic of Italy | 1.33 (0.81-2.12) | 1.08 (0.7-1.6) | -0.66 (-0.77 - -0.56) | 36.23 (21.44-59.34) | 27.51 (17.36-42.51) | -0.88 (-0.98 - -0.78) |
| Kingdom of Norway | 1.3 (0.73-2.33) | 0.87 (0.47-1.5) | -1.21 (-1.7 - -0.71) | 34.91 (18.84-65.33) | 23.32 (12.58-41.76) | -1.22 (-1.82 - -0.62) |
| Republic of Malta | 1.72 (0.96-3.16) | 1.14 (0.66-2.06) | -1.14 (-1.53 - -0.75) | 45.56 (24.4-87.54) | 28.66 (15.85-53.23) | -1.29 (-1.7 - -0.88) |
| Portuguese Republic | 1.44 (0.91-2.49) | 1.29 (0.74-1.97) | -0.41 (-0.66 - -0.16) | 41.93 (25.45-74.84) | 38.38 (21.09-61.4) | -0.41 (-0.66 - -0.15) |
| Kingdom of the Netherlands | 1.41 (0.83-2.37) | 1.07 (0.67-1.69) | -0.84 (-1.03 - -0.64) | 37 (20.62-63.87) | 27.63 (15.54-46.04) | -0.88 (-1.15 - -0.61) |
| United Kingdom of Great Britain and Northern Ireland | 2.72 (1.55-4.04) | 1.86 (1.24-2.47) | -1.19 (-1.35 - -1.03) | 71.44 (38.49-118.2) | 48.23 (28.67-68.21) | -1.22 (-1.35 - -1.09) |
| Kingdom of Sweden | 0.88 (0.59-1.25) | 0.87 (0.61-1.29) | -0.02 (-0.32 - 0.28) | 24.25 (16.24-34.66) | 22.79 (14.74-34.56) | -0.18 (-0.46 - 0.09) |
| Kingdom of Spain | 1.38 (0.96-2.09) | 0.82 (0.54-1.24) | -1.5 (-1.75 - -1.25) | 40.77 (27.78-63.4) | 23.2 (13.92-36.47) | -1.65 (-1.95 - -1.36) |
| Swiss Confederation | 1.54 (0.95-2.67) | 0.98 (0.67-1.35) | -1.45 (-1.53 - -1.36) | 42.65 (25.41-74.28) | 25.51 (16.48-38.27) | -1.48 (-1.75 - -1.2) |
| Republic of Chile | 0.75 (0.39-1.5) | 0.88 (0.47-1.51) | 0.49 (0.15 - 0.83) | 20.05 (9.84-41.39) | 23.77 (12.22-42.06) | 0.55 (0.17 - 0.93) |
| Argentine Republic | 1.57 (1.01-2.45) | 1.4 (0.92-2.17) | -0.23 (-0.54 - 0.07) | 41.81 (26.25-66.77) | 38.53 (23.51-61.8) | -0.11 (-0.43 - 0.2) |
| Canada | 1.23 (0.78-1.9) | 0.93 (0.68-1.26) | -0.84 (-1.25 - -0.44) | 33.7 (20.65-52.93) | 25.18 (18.16-33.41) | -0.87 (-1.08 - -0.65) |
| Eastern Republic of Uruguay | 1.45 (0.92-2.44) | 1.58 (1.02-2.48) | 0.25 (-0.07 - 0.57) | 40.06 (23.99-69.95) | 42.61 (27-70.28) | 0.28 (-0.04 - 0.6) |
| United States of America | 1.5 (0.94-2.55) | 1.21 (0.75-1.9) | -0.64 (-0.81 - -0.46) | 42.92 (25.97-74.49) | 34.91 (21.44-54.73) | -0.63 (-0.81 - -0.45) |
| Commonwealth of the Bahamas | 1.61 (0.96-2.87) | 2.93 (1.76-4.83) | 2.1 (1.92 - 2.28) | 48.33 (27.6-88.29) | 88.87 (51.3-148.9) | 1.89 (0.87 - 2.92) |
| Republic of Cuba | 0.72 (0.44-1.17) | 1.08 (0.69-1.71) | 0.96 (0.22 - 1.7) | 19.98 (12.11-33.28) | 28.28 (17.75-46.47) | 0.78 (0.11 - 1.46) |
| Barbados | 1.92 (1.25-3.16) | 2.92 (1.82-4.35) | 1.3 (0.9 - 1.69) | 54.71 (34.58-91.75) | 82.34 (49.59-125.02) | 1.24 (0.81 - 1.67) |
| Antigua and Barbuda | 1.32 (0.73-2.5) | 1.93 (1.1-3.27) | 1.1 (0.93 - 1.28) | 35.42 (19.32-68.09) | 58.54 (32.95-102.39) | 1.44 (1.26 - 1.62) |
| Commonwealth of Dominica | 1.27 (0.63-2.41) | 2.26 (1.19-3.92) | 1.74 (1.54 - 1.94) | 36.84 (17.45-70.38) | 66.84 (35.12-118.6) | 1.77 (1.55 - 1.99) |
| Belize | 0.53 (0.32-0.85) | 0.99 (0.6-1.57) | 1.88 (1.64 - 2.11) | 15.09 (8.74-24.44) | 29.76 (17.69-47.2) | 2.11 (1.87 - 2.35) |
| Grenada | 1.17 (0.67-2.16) | 1.68 (0.98-2.73) | 1.07 (0.82 - 1.33) | 32.78 (17.93-62.67) | 51.27 (29.37-84.03) | 1.32 (1.06 - 1.58) |
| Dominican Republic | 0.51 (0.27-1.01) | 1.14 (0.62-1.95) | 2.54 (2.24 - 2.85) | 14.44 (7.55-29.65) | 33.4 (17.4-57.23) | 2.6 (2.29 - 2.92) |
| Jamaica | 1.05 (0.74-1.53) | 1.56 (1.05-2.4) | 1.24 (0.79 - 1.69) | 31.19 (21.39-45.59) | 48.93 (31.62-78.84) | 1.45 (0.94 - 1.96) |
| Republic of Guyana | 1.12 (0.67-1.96) | 2.13 (1.25-3.27) | 1.83 (0.8 - 2.88) | 33.81 (19.92-60.66) | 65.92 (38.33-100.48) | 2.05 (1.03 - 3.07) |
| Saint Lucia | 1.86 (1-3.26) | 1.74 (1.12-2.51) | -0.28 (-0.75 - 0.19) | 57.4 (30.17-102.15) | 55.39 (34.63-80.72) | -0.18 (-0.72 - 0.36) |
| Republic of Haiti | 0.4 (0.22-0.73) | 0.82 (0.46-1.32) | 2.25 (2.07 - 2.43) | 12.17 (7-21.35) | 24.03 (13.2-38.48) | 2.16 (1.92 - 2.4) |
| Republic of Suriname | 0.85 (0.49-1.48) | 1.24 (0.75-1.95) | 1.18 (0.72 - 1.65) | 25.38 (14.56-44.33) | 38.31 (23-59.37) | 1.3 (0.75 - 1.86) |
| Saint Vincent and the Grenadines | 0.99 (0.56-1.84) | 1.5 (0.86-2.56) | 1.09 (0.12 - 2.07) | 28.05 (15.59-53.79) | 46.19 (25.78-81.26) | 1.41 (0.56 - 2.27) |
| Republic of Peru | 0.28 (0.16-0.48) | 0.75 (0.43-1.24) | 3.19 (2.75 - 3.63) | 7.92 (4.31-13.92) | 21.84 (12.16-37.5) | 3.39 (2.89 - 3.89) |
| Plurinational State of Bolivia | 0.43 (0.21-0.92) | 1.18 (0.6-2.32) | 3.08 (2.9 - 3.26) | 12.39 (6.01-27.17) | 36.92 (18.64-73.48) | 3.33 (3.19 - 3.47) |
| Republic of Trinidad and Tobago | 1.22 (0.75-2.08) | 1.72 (1.15-2.77) | 1.17 (0.64 - 1.7) | 33.5 (20.02-59.45) | 49.65 (31.75-82.03) | 1.3 (0.9 - 1.7) |
| Republic of Ecuador | 0.41 (0.23-0.81) | 0.78 (0.5-1.12) | 2.04 (1.26 - 2.83) | 11.65 (6.34-23.69) | 23.53 (15.42-35.1) | 2.24 (1.52 - 2.96) |
| Republic of Costa Rica | 0.77 (0.51-1.16) | 1.3 (0.77-2.02) | 1.76 (1.23 - 2.29) | 21.78 (14.4-32.94) | 37.05 (21.33-59.54) | 1.74 (1.18 - 2.3) |
| Republic of Colombia | 0.57 (0.36-0.91) | 0.74 (0.51-1.05) | 0.82 (0.24 - 1.4) | 15.14 (9.32-24.74) | 21.68 (15.05-31.13) | 1.13 (0.6 - 1.66) |
| Republic of Nicaragua | 0.44 (0.25-0.76) | 0.92 (0.54-1.35) | 2.39 (2.03 - 2.75) | 12.24 (6.71-21.53) | 27.16 (15.51-43.08) | 2.51 (1.93 - 3.09) |
| Republic of Honduras | 0.33 (0.17-0.61) | 0.67 (0.35-1.17) | 2.22 (2.11 - 2.33) | 9.13 (4.67-17.1) | 17.5 (8.97-31.25) | 2.04 (1.9 - 2.18) |
| Republic of El Salvador | 0.43 (0.25-0.76) | 1.26 (0.74-2.04) | 3.43 (3.07 - 3.79) | 12.64 (7.22-22.37) | 37.28 (21.33-60.48) | 3.34 (3.03 - 3.65) |
| United Mexican States | 0.96 (0.65-1.24) | 1.07 (0.82-1.35) | 0.28 (0.06 - 0.51) | 29.39 (19.07-38.28) | 32.92 (25.18-41.25) | 0.31 (0.08 - 0.55) |
| Republic of Guatemala | 0.41 (0.24-0.67) | 0.87 (0.63-1.2) | 2.38 (2.16 - 2.61) | 11.66 (6.72-19.73) | 25.86 (18.34-35.91) | 2.56 (1.62 - 3.5) |
| Republic of Panama | 0.83 (0.45-1.39) | 1.26 (0.88-1.66) | 1.27 (0.75 - 1.79) | 21.78 (11.56-37.72) | 35.99 (24.31-49.91) | 1.53 (1 - 2.07) |
| Federative Republic of Brazil | 1.46 (0.85-1.97) | 1.45 (0.86-2.02) | 0.03 (-0.25 - 0.3) | 43.62 (25.27-59.43) | 42.94 (24.24-61.9) | -0.07 (-0.24 - 0.1) |
| Bolivarian Republic of Venezuela | 0.76 (0.49-1.21) | 1.84 (1.1-2.9) | 2.64 (1.85 - 3.44) | 21.54 (13.74-34.11) | 52.08 (32.8-78.12) | 2.81 (2.49 - 3.14) |
| Kingdom of Bahrain | 1.52 (0.87-3.03) | 1.74 (1.03-2.63) | 0.37 (0.01 - 0.72) | 44.1 (24.97-90.3) | 51.16 (29.69-76.26) | 0.39 (0.13 - 0.65) |
| Republic of Paraguay | 1.13 (0.55-1.82) | 1.91 (1.02-2.93) | 1.55 (1.32 - 1.78) | 33.69 (16.23-53.13) | 53.75 (28.53-82.15) | 1.35 (1.13 - 1.57) |
| State of Kuwait | 1.78 (1.09-2.52) | 1.97 (1.41-2.62) | 0.39 (-0.8 - 1.6) | 50.55 (30.16-71.87) | 53.51 (38.49-70.6) | 0.19 (-0.88 - 1.27) |
| Republic of Iraq | 0.71 (0.41-1.24) | 2.36 (1.29-3.69) | 3.75 (3.54 - 3.97) | 22.91 (13.36-42.08) | 79.8 (44.34-123.72) | 3.9 (3.67 - 4.13) |
| Arab Republic of Egypt | 0.3 (0.18-0.56) | 1.63 (0.9-2.6) | 5.34 (4.63 - 6.06) | 9.47 (5.73-17.04) | 45.43 (26.12-72.21) | 4.98 (4.42 - 5.55) |
| People's Democratic Republic of Algeria | 0.49 (0.27-0.93) | 1.1 (0.59-1.79) | 2.49 (2.22 - 2.75) | 15.11 (7.99-28.83) | 37.48 (19.51-61.55) | 2.81 (2.54 - 3.08) |
| Islamic Republic of Iran | 0.27 (0.16-0.48) | 0.58 (0.36-0.87) | 2.24 (2.01 - 2.47) | 7.96 (4.58-13.9) | 18.35 (11.01-28.21) | 2.47 (2.24 - 2.71) |
| Hashemite Kingdom of Jordan | 1.15 (0.66-1.84) | 2.11 (1.39-2.85) | 1.83 (1.39 - 2.28) | 37.39 (21.54-60.13) | 61.93 (40.76-83.16) | 1.5 (1.06 - 1.95) |
| Lebanese Republic | 0.85 (0.48-1.48) | 2.06 (1.33-3.02) | 3.33 (2.91 - 3.76) | 24.17 (13.21-44.58) | 64.74 (39.73-94.94) | 3.38 (2.6 - 4.17) |
| Kingdom of Morocco | 0.59 (0.27-1.3) | 1.75 (0.9-2.98) | 3.36 (3.25 - 3.48) | 17.45 (8.06-38.47) | 60.5 (31.14-103.82) | 3.87 (3.74 - 3.99) |
| State of Qatar | 1.47 (0.82-2.83) | 1.73 (0.99-2.65) | 0.45 (0.11 - 0.8) | 40.3 (22.99-77.25) | 45.19 (26.83-68.01) | 0.34 (0.03 - 0.64) |
| Palestine | 0.87 (0.47-1.58) | 2.13 (1.25-3.27) | 2.77 (2.59 - 2.95) | 25.39 (13.68-47.52) | 61.51 (36.2-93.64) | 2.7 (2.58 - 2.81) |
| State of Libya | 0.45 (0.28-0.72) | 1.27 (0.7-2.07) | 3.09 (2.68 - 3.5) | 14.47 (9.01-23.22) | 41.63 (23.9-66.1) | 3.2 (2.68 - 3.72) |
| Sultanate of Oman | 0.46 (0.24-0.85) | 0.81 (0.51-1.23) | 1.9 (1.69 - 2.11) | 13.16 (6.72-24.54) | 24.14 (14.92-36.5) | 1.93 (1.73 - 2.13) |
| Kingdom of Saudi Arabia | 0.65 (0.37-1.13) | 1.98 (1.29-2.88) | 3.49 (3.13 - 3.86) | 17.56 (9.88-31) | 51.82 (33.92-76.7) | 3.39 (3.13 - 3.64) |
| Republic of Tunisia | 1.02 (0.53-2.03) | 2.48 (1.51-3.79) | 2.69 (2.51 - 2.87) | 29.71 (15.21-57.8) | 78.7 (47.03-121.51) | 2.98 (2.81 - 3.16) |
| Republic of Yemen | 0.2 (0.08-0.45) | 0.38 (0.2-0.66) | 2.04 (1.77 - 2.32) | 4.96 (2.05-11.04) | 10.66 (5.57-19.43) | 2.38 (2.11 - 2.66) |
| Syrian Arab Republic | 0.46 (0.23-0.91) | 0.94 (0.64-1.4) | 2.16 (1.71 - 2.62) | 13.83 (6.74-28.49) | 26.84 (17.44-40.12) | 2.01 (1.51 - 2.51) |
| Republic of Turkey | 0.62 (0.33-1.12) | 1 (0.6-1.45) | 1.49 (1.1 - 1.88) | 18.25 (9.92-33.05) | 30.19 (17.53-46.55) | 1.52 (1.03 - 2.02) |
| United Arab Emirates | 2.26 (1.19-4.08) | 2.33 (1.57-3.44) | 0.06 (-0.73 - 0.86) | 65.06 (34.2-116.29) | 53.15 (35.28-78.43) | -0.67 (-1.38 - 0.03) |
| Kingdom of Bhutan | 0.45 (0.2-1.04) | 0.73 (0.38-1.45) | 1.42 (1.28 - 1.56) | 14.27 (6.22-32.79) | 23.96 (12.15-49.53) | 1.55 (1.36 - 1.75) |
| Islamic Republic of Afghanistan | 0.25 (0.12-0.49) | 0.41 (0.22-0.8) | 1.55 (1.3 - 1.79) | 7.25 (3.59-15.08) | 12.93 (6.64-23.72) | 1.75 (1.5 - 2.01) |
| Republic of India | 0.42 (0.22-0.78) | 0.94 (0.5-1.6) | 2.41 (1.94 - 2.87) | 12.85 (6.83-24.87) | 27.49 (14.62-47.18) | 2.23 (1.69 - 2.78) |
| Islamic Republic of Pakistan | 1.07 (0.61-1.88) | 2.19 (1.27-3.83) | 2.15 (1.8 - 2.5) | 31.37 (18.18-55.7) | 68.93 (40.16-120.25) | 2.37 (2.02 - 2.73) |
| People's Republic of Bangladesh | 0.26 (0.12-0.52) | 0.97 (0.47-1.73) | 3.84 (3.4 - 4.28) | 9.5 (3.96-19.22) | 34.75 (15.69-62.81) | 3.84 (3.36 - 4.33) |
| Republic of Angola | 0.54 (0.27-1.07) | 1.41 (0.74-2.86) | 2.93 (2.38 - 3.49) | 19.04 (9.3-38.61) | 52.32 (27.17-107.82) | 2.99 (2.46 - 3.53) |
| Federal Democratic Republic of Nepal | 0.26 (0.15-0.44) | 0.57 (0.29-1.13) | 2.41 (2.12 - 2.69) | 7.87 (4.57-13.52) | 16.52 (8.54-32.87) | 2.25 (2.07 - 2.43) |
| Republic of the Congo | 1.29 (0.61-2.62) | 3.73 (2.05-6.5) | 3.35 (2.97 - 3.72) | 47.53 (22.55-98.3) | 134.76 (71.07-237.66) | 3.29 (2.63 - 3.95) |
| Gabonese Republic | 1.73 (0.87-3.12) | 3.93 (2.26-5.86) | 2.45 (1.74 - 3.17) | 63.51 (31.62-114.35) | 148.47 (82.56-223.54) | 2.54 (1.77 - 3.31) |
| Democratic Republic of the Congo | 1.24 (0.62-2.33) | 3.26 (1.68-6.09) | 3.01 (2.81 - 3.21) | 44.52 (21.68-85.37) | 124.71 (63.66-237.26) | 3.19 (2.97 - 3.41) |
| Central African Republic | 0.82 (0.36-1.76) | 1.44 (0.71-3.04) | 1.78 (1.43 - 2.14) | 30.01 (12.64-65.74) | 55.31 (27.39-117.8) | 1.98 (1.51 - 2.45) |
| Republic of Equatorial Guinea | 1.11 (0.53-2.18) | 4.61 (2.34-7.49) | 4.47 (4.04 - 4.9) | 39.21 (18.41-80.64) | 173.27 (84.7-280.92) | 4.64 (4.15 - 5.12) |
| Union of the Comoros | 0.13 (0.07-0.25) | 0.25 (0.14-0.46) | 1.9 (1.79 - 2.02) | 4.13 (2.09-8.19) | 7.77 (4.47-13.94) | 1.71 (1.59 - 1.82) |
| Republic of Djibouti | 0.29 (0.15-0.54) | 0.43 (0.24-0.74) | 1.32 (0.92 - 1.73) | 9.35 (4.94-18.67) | 13.77 (7.62-24.57) | 1.28 (0.79 - 1.78) |
| Federal Democratic Republic of Ethiopia | 0.21 (0.11-0.39) | 0.71 (0.41-1.22) | 3.84 (3.64 - 4.05) | 6.22 (3.22-11.87) | 21.44 (12.01-37.82) | 3.88 (3.59 - 4.16) |
| Republic of Burundi | 0.15 (0.06-0.32) | 0.28 (0.13-0.6) | 2.01 (1.88 - 2.14) | 5.09 (2.04-11.53) | 9.48 (4.45-20.36) | 1.91 (1.76 - 2.05) |
| State of Eritrea | 0.2 (0.1-0.37) | 0.42 (0.24-0.75) | 2.29 (2.16 - 2.42) | 6.73 (3.42-12.45) | 14.37 (7.93-25.3) | 2.38 (2.22 - 2.53) |
| Republic of Kenya | 0.31 (0.16-0.61) | 0.81 (0.42-1.62) | 2.89 (2.37 - 3.41) | 10.63 (5.39-21.1) | 29.37 (14.57-58.11) | 3.15 (2.79 - 3.52) |
| Republic of Malawi | 0.18 (0.07-0.39) | 0.43 (0.2-0.94) | 2.86 (2.24 - 3.5) | 6.57 (2.67-14.42) | 15.43 (6.89-34.27) | 2.71 (1.73 - 3.71) |
| Republic of Mozambique | 0.08 (0.04-0.15) | 0.1 (0.05-0.21) | 1.04 (0.79 - 1.29) | 2.14 (1-4.16) | 2.65 (1.31-5.26) | 0.72 (0.46 - 0.97) |
| Republic of Madagascar | 0.31 (0.15-0.67) | 0.54 (0.26-1.12) | 1.61 (1.22 - 2) | 10.11 (4.6-21.38) | 15.94 (7.78-32.97) | 1.45 (1.05 - 1.85) |
| Republic of Mauritius | 0.89 (0.61-1.22) | 2.52 (1.8-3.31) | 3.1 (2.46 - 3.75) | 26.29 (17.93-36) | 73.77 (52.4-98.88) | 3.07 (2.44 - 3.71) |
| Republic of Rwanda | 0.19 (0.08-0.38) | 0.37 (0.18-0.68) | 1.89 (1.04 - 2.74) | 6.75 (2.71-13.9) | 11.45 (5.35-22.18) | 1.63 (1.27 - 2) |
| Federal Republic of Somalia | 0.08 (0.04-0.17) | 0.16 (0.08-0.34) | 2.03 (1.77 - 2.3) | 2.41 (1.12-4.98) | 5.39 (2.6-11.37) | 2.38 (2.11 - 2.65) |
| Republic of Seychelles | 1.1 (0.6-1.85) | 1.87 (1.15-2.84) | 1.57 (1.33 - 1.81) | 33.47 (17.63-57.24) | 61.64 (37.6-92.86) | 1.82 (1.61 - 2.04) |
| United Republic of Tanzania | 0.2 (0.11-0.35) | 0.38 (0.2-0.73) | 1.94 (1.52 - 2.36) | 6.51 (3.56-11.25) | 11.2 (5.58-21.82) | 1.7 (1.25 - 2.14) |
| Republic of Zambia | 0.63 (0.35-1.19) | 1.67 (0.97-2.92) | 2.98 (2.55 - 3.42) | 19.55 (10.5-37.91) | 53.99 (31.31-95.88) | 3.14 (2.73 - 3.55) |
| Republic of Uganda | 0.3 (0.18-0.52) | 0.86 (0.49-1.46) | 3.16 (2.96 - 3.35) | 9.92 (5.35-17.51) | 32.28 (18.36-53.81) | 3.62 (3.4 - 3.85) |
| Republic of Botswana | 0.2 (0.1-0.4) | 0.6 (0.33-1.19) | 3.5 (2.69 - 4.32) | 6.36 (3.1-12.85) | 19.97 (10-42.56) | 3.65 (2.74 - 4.57) |
| Kingdom of Lesotho | 0.42 (0.21-0.82) | 0.72 (0.4-1.37) | 1.72 (1.22 - 2.21) | 11.65 (5.78-23.16) | 20.7 (11.39-39.72) | 1.83 (1.22 - 2.45) |
| Republic of South Africa | 0.73 (0.44-1.32) | 1.72 (1.08-2.4) | 2.74 (2.13 - 3.36) | 20.6 (12.03-38.7) | 50.55 (31.45-73.66) | 2.83 (2.31 - 3.34) |
| Republic of Namibia | 0.57 (0.29-1.27) | 1.53 (0.81-2.95) | 3 (2.11 - 3.91) | 20.97 (10.56-45.89) | 55.46 (26.16-107.83) | 2.91 (1.81 - 4.02) |
| Kingdom of Eswatini | 0.74 (0.38-1.43) | 1.13 (0.56-2.23) | 1.25 (0.44 - 2.06) | 27.66 (14.02-54.3) | 40.96 (19.26-82.79) | 1.18 (0.29 - 2.09) |
| Republic of Zimbabwe | 0.25 (0.14-0.49) | 0.68 (0.35-1.39) | 3.29 (2.41 - 4.18) | 6.45 (3.36-12.84) | 20.17 (9.98-41.52) | 3.96 (3.6 - 4.32) |
| Republic of Benin | 0.29 (0.14-0.58) | 0.6 (0.3-1.17) | 2.03 (1.44 - 2.61) | 9.01 (4.33-18.01) | 20.34 (10.13-40.03) | 2.32 (2.03 - 2.61) |
| Republic of Cameroon | 0.6 (0.34-1.13) | 2.06 (1.18-3.78) | 3.76 (3.49 - 4.02) | 17.42 (9.86-33.66) | 63.45 (36.14-119.35) | 3.99 (3.7 - 4.29) |
| Burkina Faso | 0.25 (0.11-0.57) | 0.35 (0.14-0.73) | 1.2 (0.65 - 1.76) | 5.63 (2.51-12.94) | 8.81 (3.77-17.77) | 1.54 (1 - 2.09) |
| Republic of Cabo Verde | 0.54 (0.27-1.09) | 0.91 (0.52-1.72) | 1.64 (0.97 - 2.31) | 14.74 (7.34-30.99) | 27.29 (15.32-53.21) | 1.87 (1.11 - 2.63) |
| Republic of Côte d'Ivoire | 0.87 (0.4-1.91) | 2.21 (1.03-4.74) | 3 (2.55 - 3.47) | 24.9 (10.98-57.01) | 75.08 (33.23-161.17) | 3.48 (2.86 - 4.1) |
| Republic of Chad | 0.92 (0.45-1.77) | 1.3 (0.64-2.68) | 1.08 (0.91 - 1.25) | 26.45 (12.54-50.38) | 39.27 (18.57-82.33) | 1.27 (1.02 - 1.51) |
| Republic of the Gambia | 0.51 (0.26-1.01) | 1.34 (0.69-2.57) | 3.07 (2.65 - 3.49) | 15.97 (8.01-32.65) | 45.12 (22.16-88.13) | 3.11 (2.52 - 3.7) |
| Republic of Guinea | 0.39 (0.18-0.78) | 0.65 (0.34-1.29) | 1.61 (1.39 - 1.84) | 11.36 (5.33-22.81) | 20.86 (10.5-42.5) | 1.89 (1.57 - 2.21) |
| Republic of Liberia | 0.71 (0.36-1.51) | 2.15 (1.04-4.49) | 3.4 (2.89 - 3.9) | 22.12 (10.64-48.4) | 74.39 (35.07-162.98) | 3.76 (3.29 - 4.24) |
| Republic of Ghana | 0.93 (0.45-1.65) | 1.81 (1.01-3.05) | 2.11 (1.79 - 2.43) | 29.5 (14.1-53) | 60.69 (33.07-104.62) | 2.27 (1.92 - 2.61) |
| Republic of Guinea-Bissau | 0.45 (0.22-1.06) | 1.22 (0.57-2.84) | 3.06 (2.42 - 3.69) | 13.04 (6.29-31.01) | 37.79 (17.45-90.71) | 3.01 (2.71 - 3.31) |
| Republic of Mali | 1.15 (0.62-2.24) | 2.86 (1.24-5.16) | 2.81 (2.21 - 3.4) | 39.44 (20.67-79.12) | 107.02 (43.91-197.95) | 3.09 (2.47 - 3.71) |
| Republic of the Niger | 0.62 (0.31-1.27) | 0.98 (0.48-2.11) | 1.44 (1.14 - 1.73) | 17.59 (8.37-37.37) | 29.42 (14.05-64.4) | 1.62 (1.28 - 1.95) |
| Islamic Republic of Mauritania | 1.08 (0.55-1.95) | 3.13 (1.82-5.15) | 3.33 (2.67 - 3.99) | 30.62 (14.7-61.72) | 104.89 (59.31-175.2) | 3.85 (3.14 - 4.57) |
| Federal Republic of Nigeria | 0.65 (0.26-1.58) | 2 (0.83-4.15) | 3.44 (3.13 - 3.74) | 20.13 (8.42-49.95) | 60.89 (25.2-133.15) | 3.38 (3.03 - 3.74) |
| Republic of Senegal | 0.73 (0.37-1.37) | 1.73 (0.97-3.18) | 2.94 (1.92 - 3.97) | 24.22 (11.92-46.68) | 58.3 (32.37-108.9) | 2.59 (1.92 - 3.27) |
| Democratic Republic of Sao Tome and Principe | 0.34 (0.18-0.66) | 1.35 (0.69-2.54) | 4.28 (3.86 - 4.71) | 9.56 (5.18-19) | 42.9 (21.74-83.64) | 4.63 (4.19 - 5.08) |
| Republic of Sierra Leone | 0.45 (0.21-1.02) | 0.87 (0.44-1.83) | 2.07 (1.75 - 2.4) | 13.25 (6.19-30.88) | 27.19 (13.69-56.91) | 2.22 (1.94 - 2.51) |
| Togolese Republic | 0.56 (0.26-1.23) | 1.25 (0.61-2.56) | 2.48 (1.98 - 2.99) | 14.72 (6.98-33.75) | 36.64 (17.84-78.56) | 2.81 (2.26 - 3.36) |
| Bermuda | 1 (0.58-1.73) | 1.23 (0.84-1.83) | 0.66 (0.43 - 0.89) | 24.52 (13.79-44.19) | 30.79 (20.23-47.39) | 0.66 (0.44 - 0.87) |
| Greenland | 1.88 (0.99-3.53) | 1.69 (0.96-3.05) | -0.33 (-0.62 - -0.05) | 48.67 (25.58-94.66) | 38.41 (21.79-70.37) | -0.73 (-1.01 - -0.45) |
| American Samoa | 2.18 (1.42-3.41) | 3.77 (2.39-5.66) | 1.68 (1.47 - 1.89) | 61.51 (41.77-91.98) | 110.26 (68.73-160.39) | 1.84 (1.5 - 2.18) |
| Cook Islands | 2 (1.09-3.6) | 5.68 (3.54-8.7) | 3.31 (3.06 - 3.56) | 64.82 (34.39-116.74) | 165.36 (105.42-253.43) | 2.9 (2.73 - 3.07) |
| Guam | 0.83 (0.47-1.44) | 1.57 (0.87-2.32) | 2 (-0.96 - 5.05) | 23.04 (12.54-41.25) | 49.94 (26.47-74.71) | 2.36 (-0.59 - 5.4) |
| Principality of Monaco | 1.93 (0.97-4.07) | 2.65 (1.26-5.09) | 0.96 (0.81 - 1.12) | 51.65 (24.51-113.69) | 67.33 (30.95-135.84) | 0.81 (0.7 - 0.92) |
| Republic of Niue | 1.62 (0.89-3.24) | 3.29 (2.07-4.96) | 2.24 (2.13 - 2.35) | 50.1 (26.33-102.6) | 101.37 (63.5-150.67) | 2.22 (2.09 - 2.34) |
| Republic of Nauru | 3.06 (1.66-5.46) | 6.31 (3.52-10.58) | 2.23 (2.04 - 2.42) | 96.14 (52.71-172.6) | 200.67 (112.87-335.25) | 2.27 (2.07 - 2.46) |
| Northern Mariana Islands | 2.1 (1.18-3.63) | 3.11 (1.95-4.74) | 1.22 (1.02 - 1.42) | 57.3 (32.35-96.84) | 86.28 (54.77-126.24) | 1.24 (1.03 - 1.45) |
| Puerto Rico | 1.55 (0.91-2.28) | 1.31 (0.95-1.7) | -0.68 (-0.82 - -0.54) | 48.3 (27.27-73.06) | 39.31 (27.42-51.51) | -0.76 (-0.9 - -0.63) |
| Republic of Palau | 2.79 (1.6-5.05) | 4.68 (2.74-7.57) | 1.57 (1.35 - 1.8) | 76.87 (41.36-141.63) | 129.91 (75.39-210.08) | 1.59 (1.4 - 1.79) |
| Saint Kitts and Nevis | 1.59 (0.94-2.9) | 1.96 (1.2-3.13) | 0.64 (0.15 - 1.13) | 45.29 (26.57-85.2) | 58.14 (34.96-95.15) | 0.77 (0.45 - 1.08) |
| Tokelau | 1.4 (0.82-2.54) | 2.72 (1.71-4.15) | 2.15 (1.92 - 2.38) | 36.08 (22.12-61.19) | 68.62 (43.63-102.74) | 2.09 (1.84 - 2.35) |
| Republic of San Marino | 1.03 (0.53-2.17) | 0.97 (0.49-1.81) | -0.21 (-0.47 - 0.05) | 26.82 (13.3-57.53) | 29.01 (14.05-55.31) | 0.22 (-0.03 - 0.48) |
| United States Virgin Islands | 1.89 (0.95-3.75) | 1.74 (0.86-2.81) | -0.42 (-0.96 - 0.12) | 48.13 (22.6-95.97) | 53.76 (25.65-86.78) | 0.17 (-0.24 - 0.59) |
| Republic of South Sudan | 0.23 (0.11-0.49) | 0.41 (0.21-0.84) | 1.71 (1.35 - 2.07) | 7.67 (3.72-16.1) | 13.72 (6.57-28.25) | 1.85 (1.4 - 2.29) |
| Tuvalu | 1.14 (0.62-2.15) | 2.56 (1.44-4.34) | 2.5 (2.39 - 2.61) | 33.26 (17.14-65.77) | 81.23 (44.87-139.49) | 2.72 (2.6 - 2.84) |
| Republic of Sudan | 0.4 (0.18-0.84) | 0.88 (0.45-1.65) | 2.47 (2.26 - 2.68) | 11.91 (5.43-26.39) | 29.03 (14.85-55.2) | 2.73 (2.43 - 3.03) |
