# Supplementary material for: The global burden of female breast cancer attributable to high fasting plasma glucose: A comprehensive analysis of the GBD database from 1990 to 2023
Source: PLoS One. 2026 Oct 21;21(10):e0359268. doi: 10.1371/journal.pone.0359268 (PMC13686481; doi:10.1371/journal.pone.0359268)
Supplement: Supplementary file 3 [file pone.0359268.s004.docx]

Thank you very much for your thoughtful and constructive comments on our manuscript. We sincerely appreciate the time and effort you have devoted to reviewing our work. Your insightful suggestions have been invaluable in helping us improve the quality of this paper. Below, we provide a point-by-point response to your comments.

**Reviewer’s Comment 1**:“The objectives of the study are well-defined, relevant, and address a critical gap in understanding the global burden of female breast cancer caused by HFPG. While the focus on HFPG is justified, future research could expand the scope to include other risk factors (Obesity, physical inactivity, dietary habits, genetic predisposition) and molecular subtypes to provide a more comprehensive understanding of breast cancer etiology and prevention strategies.”

**Response:** Thank you for your recognition of the study’s relevance and for this excellent suggestion. We fully agree that expanding the scope to include other risk factors (such as obesity, physical inactivity, dietary habits, and genetic predisposition) and exploring associations with molecular subtypes (e.g., HER2-positive, triple-negative) would provide a more holistic understanding of breast cancer etiology. We have added a paragraph in the Discussion section (Section 4) acknowledging this limitation and explicitly recommending that future studies incorporate multi-risk-factor analyses and molecular subtype stratification when data become available.

The revised text now reads:

“Looking to the future, it is necessary to integrate additional metabolic risk factors—such as obesity, physical inactivity, and dietary patterns—alongside HFPG, and explore their interactions with breast cancer molecular subtypes. Such efforts will be crucial for developing personalized prevention strategies.”

**Reviewer’s Comment 2:** “The study has several strengths, including its comprehensive scope, use of reliable data, and focus on a significant public health issue. However, it could be improved by addressing its narrow focus, exploring molecular subtypes (e.g. HER2-Positive, triple negative), incorporating additional biomarkers (e.g. HbA1C for diabetes), and providing more detailed policy recommendations.”

**Response:** We greatly appreciate your balanced assessment. As noted above, we have now explicitly discussed this limitation and called for broader, multi-factorial approaches in future work. Regarding molecular subtypes and additional biomarkers (e.g., HbA1c): We completely agree that HbA1c is a more stable indicator of long-term glycemic control than fasting plasma glucose alone. Unfortunately, the GBD database currently does not provide stratified data by molecular subtype or HbA1c levels.

**Reviewer’s Comment 3**: “While the study mentions that ethical approval was not required, it would be helpful to explicitly state that the data used was anonymized and publicly available in the ethics section.”

**Response:**Thank you for this practical suggestion. We have revised the Ethics approval and consent to participate section (Section 2.2) to explicitly state that all data used in this study are fully anonymized and publicly available from the GBD database. The revised text now reads:

“The GBD 2023 project is an open-access database in which all data are de-identified and publicly available. The University of Washington’s Institutional Review Board reviewed and approved an informed consent waiver (https://www.Healthdata.org/research-analysis/gbd). No additional ethical approval was required for this secondary analysis of anonymized, publicly available data.”

We believe these revisions adequately address all of your concerns. Once again, we thank you for your thorough review and valuable feedback, which have significantly strengthened our manuscript. We look forward to your further consideration.
