# Supplementary material for: The global burden of female breast cancer attributable to high fasting plasma glucose: A comprehensive analysis of the GBD database from 1990 to 2023
Source: PLoS One. 2026 Oct 21;21(10):e0359268. doi: 10.1371/journal.pone.0359268 (PMC13686481; doi:10.1371/journal.pone.0359268)
Supplement: Supplementary file 4 [file pone.0359268.s006.docx]

# Response to Reviewers

Manuscript Title: The global burden of female breast cancer attributable to high fasting plasma glucose: A comprehensive analysis of the GBD database from 1990 to 2023

Dear Editor and Reviewers,

We thank you and the reviewers for the time and effort devoted to our manuscript. In this revised manuscript (page and line numbers are provided; line numbers restart on each page), we have carefully addressed every comment. All changes are highlighted in red in the revised manuscript, and point-by-point responses are provided below; page and line numbers in each response refer to the revised marked manuscript. Reviewer comments are shown in italics, followed by our responses.

Sincerely,

Huadi Shi, Zhi Song, Yaoyao Chen, Siyuan Liu, Yuling Song (on behalf of all authors)

## Response to Reviewer #1

*The authors expressed sincere appreciation for the reviewers' feedback and provided a detailed, respectful, and constructive point-by-point response... Conclusion: The authors have addressed the reviewer's comments thoroughly and professionally.*

Response: We sincerely thank the reviewer for the positive evaluation of our previous revisions. In the current revision, we have further strengthened the manuscript in response to Reviewer #2’s comments, including a systematic revision of causal-attribution language (Title, p. 1, lines 1–2; throughout), explicit definitions of the exposure metric (Methods 2.1, p. 4, lines 10–25), formal statistical analyses of the SDI–burden association (Methods 2.3, p. 5, lines 15–28; Results 3.4, p. 10, lines 1–25), and an out-of-sample validation of the projection model (Methods 2.3, p. 5, line 29 to p. 6, line 14; Results 3.5, p. 11, line 25 to p. 12, line 5). The ethics statement has also been expanded (Methods 2.2, p. 4, line 26 to p. 5, line 2), the HbA1c limitation acknowledged (Discussion, Limitations, p. 15, lines 19–23), and future multi-risk-factor and molecular-subtype research recommended (p. 15, lines 23–28).

## Response to Reviewer #2

### General Response

*Unfortunately, the current submission still suffers from significant methodological opacity, conceptual overreach, and a lack of statistical validation for its projections...*

Response: We thank the reviewer for this rigorous and constructive critique. We fully accept the core point: a GBD-based attributable-burden analysis is a descriptive, ecological assessment and must not be framed as causal inference or validated prediction. In this revision we have: (1) replaced causal wording with attribution language throughout, including the title (p. 1, lines 1–2); (2) reframed prevention-related conclusions as hypotheses for future research (Abstract Conclusion, p. 2, lines 11–18; Conclusion, p. 16, lines 9–15); (3) explicitly defined the exposure metric, HFPG above the Theoretical Minimum-Risk Exposure Level (TMREL), and distinguished it from diabetes (Methods 2.1, p. 4, lines 10–20); (4) justified the restriction to female breast cancer and removed the “systematic analysis” label (p. 4, lines 21–25; Abstract Methods, p. 1, lines 7–15); (5) added discussion of the lag-time effect and demographic drivers of the absolute burden (Results 3.1, p. 6, line 21 to p. 7, line 7; Discussion, p. 14, lines 15–27); (6) performed formal correlation analyses of the SDI–burden association and replaced “developing regions” with SDI quintile terminology (Results 3.4, p. 10, lines 1–25); and (7) performed an out-of-sample validation of the BAPC model, added an explicit statement that 2024–2050 values are model-based projections, and discussed how emerging metabolic therapies may affect long-term forecasts (Methods 2.3, p. 5, line 29 to p. 6, line 14; Results 3.5, p. 11, line 25 to p. 12, line 5; Discussion, Limitations, p. 15, line 29 to p. 16, line 7).

## Comment 1a. Conceptual and causal overreach — “caused by”

*"The manuscript repeatedly claims breast cancer is 'caused by' HFPG... The authors must replace 'caused by' with 'attributable to' throughout the text, including the title."*

Response: We fully agree. The GBD comparative risk assessment quantifies attributable burden using population exposure distributions and pooled relative risks from prospective studies; it does not establish causality within our own analysis. We have replaced "caused by" with "attributable to" throughout, including the title, which now reads: "The global burden of female breast cancer attributable to high fasting plasma glucose: A comprehensive analysis of the GBD database from 1990 to 2023" (p. 1, lines 1–2). All corresponding instances in the Abstract (p. 1, line 16 to p. 2, line 10), Results 3.3 (p. 9, lines 13–29), Discussion (p. 13, line 2), and Conclusion (p. 16, lines 9–15) have been revised accordingly.

Changes made: Title (p. 1, lines 1–2); Abstract (p. 1, line 16 to p. 2, line 10); Results 3.3 (p. 9, lines 13–29); Discussion (p. 13, line 2); Conclusion (p. 16, lines 9–15).

## Comment 1b. Prevention recommendations exceed the evidence

*"...the conclusion that these findings highlight an 'urgency' for blood-glucose control as a breast cancer prevention strategy exceeds the evidence... framing as hypotheses for further investigation."*

Response: We agree completely. As an ecological, population-level analysis, our study cannot demonstrate that glycemic intervention reduces breast cancer incidence. We have removed the word "urgency" and rewritten the Conclusion (and the corresponding sentences in the Abstract) to frame glycemic management and metabolic risk-factor control as considerations within comprehensive prevention frameworks and as hypotheses for future research, explicitly stating that "as this is an ecological, population-level analysis, whether glucose-lowering interventions can reduce breast cancer incidence remains to be established in prospective interventional studies." Prevention-oriented recommendations in the Discussion (p. 14, line 28 to p. 15, line 14) have likewise been framed as suggestions for high-burden regions rather than claims of established benefit.

Changes made: Abstract Conclusion (p. 2, lines 11–18); Conclusion (p. 16, lines 9–15); Discussion recommendations (p. 14, line 28 to p. 15, line 14).

## Comment 2a. HFPG must be explicitly defined, including TMREL; HFPG is not diabetes

*"The authors treat HFPG interchangeably with diabetes. They must explicitly define the GBD exposure metric, specifically the Theoretical Minimum-Risk Exposure Level (TMREL)..."*

Response: Thank you for this important comment. We have added an explicit definition in Methods 2.1 (p. 4, lines 10–20): HFPG is defined as fasting plasma glucose exceeding the Theoretical Minimum-Risk Exposure Level (TMREL), approximately 4.8–5.4 mmol/L (86.4–97.2 mg/dL) in the GBD 2023 risk factor analysis. We now state explicitly that (1) the burden estimates represent the fraction of female breast cancer burden attributable to HFPG under the TMREL counterfactual, as modeled by GBD using pooled relative risks from prospective cohort studies; and (2) HFPG is a continuous exposure above the TMREL and is not equivalent to a clinical diagnosis of diabetes mellitus, capturing the full spectrum of elevated fasting glucose including prediabetic ranges. In addition, where diabetes studies are cited in the Introduction, we added transitional wording (p. 3, lines 8–20) clarifying that they provide supportive evidence for a related but narrower exposure construct; a parallel caveat was added at the end of the mechanistic Discussion paragraph (p. 12, line 26 to p. 13, line 13) and in the Limitations (p. 15, lines 19–23) noting that our estimates are not directly comparable with studies of diabetes-attributable breast cancer burden.

Changes made: Methods 2.1, new definitional paragraph (p. 4, lines 10–20); Introduction transitional wording (p. 3, lines 8–20); Discussion mechanism caveat (p. 12, line 26 to p. 13, line 13); Limitations (p. 15, lines 19–23).

## Comment 2b. Justify the restriction to female breast cancer

*"The analysis is restricted to 'female' breast cancer without justification..."*

Response: We have clarified this in Methods 2.1 (p. 4, lines 21–25): the analysis was restricted to female breast cancer because the GBD comparative risk assessment models HFPG as a risk factor for female breast cancer only; no risk–outcome pair between HFPG and male breast cancer has been established in the GBD framework, owing to the rarity of male breast cancer and the limited evidence base for quantitative risk estimation. This is a data-availability constraint rather than a design choice, and we now state this explicitly.

Changes made: Methods 2.1 (p. 4, lines 21–25).

## Comment 2c. "‘Systematic Analysis’" is misleading

*"‘Systematic Analysis’? This is a secondary analysis of a public database..."*

Response: We agree and have removed this label. The Abstract Methods now describes the study as "a comprehensive descriptive analysis of the burden of female BC-HFPG based on the GBD 2023 database" (p. 1, lines 7–15). (Reference titles containing "a systematic analysis," which refer to the GBD collaborators’ own papers, are retained as published.)

Changes made: Abstract Methods (p. 1, lines 7–15); Methods 2.3 (p. 5, lines 15–28).

## Comment 3. Lag-time effect, trend decomposition, and absolute numbers vs. age-standardized rates

*"The authors attributed the rise in ASMR/ASDR solely to HFPG... lag-time effect... decompose the trend... conflate absolute numbers (48,307 deaths) with age-standardized rates."*

Response: We thank the reviewer for this insightful comment and have addressed it in three ways:

(1) Absolute vs. age-standardized burden. Results 3.1 now explicitly reports the divergence (p. 6, line 21 to p. 7, line 7): the absolute number of BC-HFPG deaths increased by 150.0% (from 19,328 in 1990 to 48,307 in 2023), whereas the ASMR rose by only 8.7% (from 0.92 to 1.00 per 100,000). We state that this divergence indicates the growth in absolute burden is largely driven by population growth and demographic aging, whereas the modest rise in age-standardized rates reflects an increase in individual-level metabolic risk beyond demographic change.

(2) Lag-time effect. A new Discussion paragraph (p. 14, lines 15–27) acknowledges that oncogenesis following metabolic exposure involves a substantial lag period; the burden observed in any given year partly reflects HFPG exposure from years to decades earlier, so recent changes in diabetes screening and management will only become visible in BC-HFPG trends after a delay.

(3) Trend decomposition. The same paragraph (p. 14, lines 15–27) acknowledges that aggregate GBD estimates do not permit formal decomposition of the trends into changing HFPG prevalence, breast cancer survival, diagnostic intensity, and population aging; the divergent trajectories of counts versus rates suggest demographic change is the dominant driver of the absolute burden, and we call for future studies employing formal decomposition methods (e.g., age-period-cohort decomposition).

Changes made: Results 3.1 (p. 6, line 21 to p. 7, line 7); Discussion, new paragraph on temporal-trend interpretation (p. 14, lines 15–27).

## Comment 4. Ecological fallacy of the SDI association; "developing regions" is not a formal GBD classification

*"The claim that SDI and BC-HFPG burden are linked is currently descriptive and prone to the Ecological Fallacy... A formal regression analysis or correlation coefficient (accounting for regional dependence) is required... use SDI quintiles..."*

Response: We agree, and have made the following revisions:

(1) Formal correlation analyses (Methods 2.3, p. 5, lines 15–28; Results 3.4, p. 10, lines 1–25). Pearson and Spearman correlation coefficients with 95% CIs from 1,000 bootstrap resamples were calculated between the 2023 SDI and ASMR/ASDR across all 204 countries and territories. SDI showed a weak positive correlation with ASMR (Pearson r = 0.13, 95% CI: 0.02–0.24, P = 0.073; Spearman ρ = 0.20, 95% CI: 0.06–0.32, P = 0.004) and no significant correlation with ASDR (Pearson r = 0.03, 95% CI: −0.08 to 0.15; Spearman ρ = 0.09, 95% CI: −0.04 to 0.24, P = 0.196). To account for regional dependence, a sensitivity analysis at the level of the 21 GBD regions showed that the correlations attenuated and reversed direction (ASMR: r = −0.37, P = 0.101; ASDR: r = −0.46, P = 0.036) (new Fig. 4, caption at p. 11, lines 13–17). LOESS curves revealed a non-linear, inverted-U-shaped relationship peaking at an SDI of approximately 0.75 (new Fig. 3, caption at p. 11, lines 4–12). These coefficients are interpreted strictly as ecological associations.

(2) SDI quintile terminology. "Developing regions" and similar informal terms have been replaced with GBD’s formal SDI quintile classification throughout (Abstract Conclusion, p. 2, lines 11–18; Discussion, p. 13, line 18 to p. 14, line 14).

(3) Quantitative evidence across SDI quintiles (Results 3.4, p. 10, lines 17–25). In 2023, both ASMR and ASDR were highest in the low-middle SDI quintile (ASMR 1.22, 95% UI: 0.70–2.12; ASDR 37.7, 95% UI: 21.7–67.5 per 100,000), with statistically significant inter-quintile differences (Kruskal–Wallis H = 29.9 for ASMR and 25.7 for ASDR, both P < 0.001). The low-SDI quintile had a 1.18-fold higher ASDR than the high-SDI quintile (32.6 vs. 27.6 per 100,000), whereas ASMR was comparable (0.98 vs. 0.98 per 100,000). The revised text states that the burden is non-linearly distributed across the development spectrum, peaking in low-middle SDI countries, rather than making a blanket claim about "developing regions."

Changes made: Methods 2.3 (p. 5, lines 15–28); Results 3.4 (p. 10, lines 1–25) with new Figs. 2–4 (Fig. 2 caption p. 10, line 26 to p. 11, line 3; Fig. 3 caption p. 11, lines 4–12; Fig. 4 caption p. 11, lines 13–17); Abstract Conclusion (p. 2, lines 11–18); Discussion (p. 13, line 18 to p. 14, line 14).

## Comment 5a. Out-of-sample validation of the BAPC model

*"The Bayesian age-period-cohort (BAPC) model is a 'black box'... provide out-of-sample validation (e.g., training the model on 1990–2015 data to see if it accurately predicts the observed 2016–2023 GBD estimates)."*

Response: We agree that model validation is essential. We performed a temporal out-of-sample validation: the BAPC model was trained on 1990–2015 data and used to retroactively predict ASMR and ASDR for the held-out 2016–2023 period, which were compared with observed GBD 2023 estimates. The procedure is described in Methods 2.3 (p. 5, line 29 to p. 6, line 14), and the results in Results 3.5 (p. 11, line 25 to p. 12, line 5) with the observed-versus-predicted comparison shown in new Fig. 6 (caption at p. 12, lines 14–18). Predictive accuracy was high: MAPE = 1.88% for ASMR (RMSE = 0.051 per 100,000) and 2.06% for ASDR (RMSE = 1.485 per 100,000), and all eight held-out observations fell within the corresponding 95% prediction intervals (coverage = 100%). Notably, prediction errors increased monotonically with the forecast horizon, from below 1.3% for one- to five-year-ahead predictions to approximately 6% by year eight, and the model systematically under-predicted the accelerated burden observed in 2021–2023. These findings underpin an explicit statement in Results 3.5 that longer-range projections, including those to 2050, carry substantial uncertainty and should be interpreted with caution.

Changes made: Methods 2.3 (p. 5, line 29 to p. 6, line 14); Results 3.5 (p. 11, line 25 to p. 12, line 5); new Fig. 6 (caption at p. 12, lines 14–18).

## Comment 5b. Projection starting in 2024

*"The projection begins in 2024, yet the current evaluation date is August 2026..."*

Response: We clarify that official GBD estimates are currently published through 2023 only (GBD 2023 round); no empirical GBD estimates exist for 2024 or later, so our projection period does not overlap with any published empirical estimates. All values presented for 2024–2050 are model-based projections generated by the BAPC model and do not represent observed estimates; this is now stated explicitly in Methods 2.3 (p. 6, lines 11–14) and in the Fig. 5 legend (p. 12, lines 6–13).

Changes made: Methods 2.3 (p. 6, lines 11–14); Results 3.5 (p. 11, line 18 to p. 12, line 5); Fig. 5 legend (p. 12, lines 6–13).

## Comment 5c. Long-term projections do not account for changing metabolic management (GLP-1 receptor agonists)

*"The 2050 linear projection fails to account for the current global shift in metabolic management (e.g., the rapid uptake of GLP-1 receptor agonists)..."*

Response: We agree. The Limitations now note that our projections to 2050 assume historical exposure and mortality trends continue unchanged, whereas the rapid global uptake of novel metabolic therapies—most notably GLP-1 receptor agonists—together with expanding diabetes screening and management programmes could substantially alter future HFPG exposure trajectories. If these interventions translate into sustained population-level reductions in fasting glucose, the projected rise in BC-HFPG burden may be overestimated; our forecasts should be interpreted as a reference scenario conditional on the continuation of current trends rather than a prediction of an inevitable future (p. 15, line 29 to p. 16, line 7).

Changes made: Discussion, Limitations (p. 15, line 29 to p. 16, line 7).

## Minor Comment 1. Inconsistency between 1992–2023 and 1990–2023

*"The Results state a trend analysis from 1992–2023, yet the reported values and Table 1 refer to 1990–2023..."*

Response: We apologize for this typographical inconsistency. All trend descriptions now uniformly refer to 1990–2023, consistent with Table 1 (p. 7, line 8 to p. 8) and the AAPC estimates (Abstract Results, p. 1, line 16 to p. 2, line 10; Results 3.1, p. 6, line 21 to p. 7, line 7).

Changes made: Abstract Results (p. 1, line 16 to p. 2, line 10); Results 3.1 (p. 6, line 21 to p. 7, line 7).

## Minor Comment 2. AAPC confidence intervals in the text

*"Average Annual Percent Changes (AAPC) are reported as 0.28% and 0.44%. The authors must provide 95% Confidence Intervals (CIs) in the text..."*

Response: We now report the AAPCs with 95% CIs in both the Abstract and the Results text: the ASMR AAPC was 0.28% (95% CI: 0.18–0.38%) and the ASDR AAPC was 0.44% (95% CI: 0.37–0.51%). Both confidence intervals exclude zero, indicating statistically significant increasing trends (Abstract Results, p. 1, line 16 to p. 2, line 10; Results 3.1, p. 6, lines 27–29 and p. 7, lines 1–2).

Changes made: Abstract Results (p. 1, line 16 to p. 2, line 10); Results 3.1 (p. 6, lines 27–29 to p. 7, lines 1–2).

## Minor Comment 3. Uncertainty intervals in Figures 2 and 3

*"Figures 2 and 3 must include uncertainty intervals to prevent an appearance of false precision in the long-term forecasts."*

Response: We have revised all forecast-related figures to display their underlying uncertainty. The forecast figure (Fig. 5, p. 12, lines 6–13) now shows 95% uncertainty intervals for the observed period (1990–2023) and 95% prediction intervals for the projected period (2024–2050), with the legend specifying the meaning of the lines and shaded bands and stating explicitly that 2024–2050 values are model-based projections rather than observed estimates. The regional frontier figure (Fig. 2, p. 10, line 26 to p. 11, line 3) now displays 95% uncertainty intervals of the GBD estimates as error bars at 5-year intervals, and the SDI–burden association figure (Fig. 3, p. 11, lines 4–12) shows LOESS fits with 95% confidence bands. In addition, the out-of-sample validation (Fig. 6, p. 12, lines 14–18) provides direct empirical evidence of forecast uncertainty: prediction errors grow from <1.3% at short horizons to ≈6% at eight years, which we cite when urging caution about long-range extrapolations (p. 11, line 25 to p. 12, line 5).

Changes made: Fig. 5 and legend (p. 12, lines 6–13); Fig. 2 and legend (p. 10, line 26 to p. 11, line 3); Fig. 3 and legend (p. 11, lines 4–12); Fig. 6 and legend (p. 12, lines 14–18); Results 3.5 (p. 11, line 25 to p. 12, line 5).

# Closing

We hope that these revisions have fully addressed the reviewers’ concerns. We are grateful for the opportunity to improve our manuscript and welcome any further suggestions.

Sincerely,

Huadi Shi, Zhi Song, Yaoyao Chen, Siyuan Liu, Yuling Song

On behalf of all authors
