# Supplementary material for: The global burden of female breast cancer attributable to high fasting plasma glucose: A comprehensive analysis of the GBD database from 1990 to 2023
Source: PLoS One. 2026 Oct 21;21(10):e0359268. doi: 10.1371/journal.pone.0359268 (PMC13686481; doi:10.1371/journal.pone.0359268)
Supplement: Peer review history

# Peer review: Decision Letter 0

**DOI:** 10.1371/journal.pone.0359268.r001

**Authors:** Pengpeng Ye

22 Jun 2026

Dear Dr. Song,

Thank you for submitting your manuscript to PLOS ONE. After careful consideration, we feel that it has merit but does not fully meet PLOS ONE’s publication criteria as it currently stands. Therefore, we invite you to submit a revised version of the manuscript that addresses the points raised during the review process.

Please submit your revised manuscript by Aug 06 2026 11:59PM. If you will need more time than this to complete your revisions, please reply to this message or contact the journal office at plosone@plos.org. When you're ready to submit your revision, log on to https://www.editorialmanager.com/pone/ and select the 'Submissions Needing Revision' folder to locate your manuscript file.

- A letter that responds to each point raised by the academic editor and reviewer(s). You should upload this letter as a separate file labeled 'Response to Reviewers'.
- A marked-up copy of your manuscript that highlights changes made to the original version. You should upload this as a separate file labeled 'Revised Manuscript with Track Changes'.
- An unmarked version of your revised paper without tracked changes. You should upload this as a separate file labeled 'Manuscript'.

If you would like to make changes to your financial disclosure, please include your updated statement in your cover letter. Guidelines for resubmitting your figure files are available below the reviewer comments at the end of this letter.

If applicable, we recommend that you deposit your laboratory protocols in protocols.io to enhance the reproducibility of your results. Protocols.io assigns your protocol its own identifier (DOI) so that it can be cited independently in the future. For instructions see: https://journals.plos.org/plosone/s/submission-guidelines#loc-laboratory-protocols. Additionally, PLOS ONE offers an option for publishing peer-reviewed Lab Protocol articles, which describe protocols hosted on protocols.io. Read more information on sharing protocols at https://plos.org/protocols?utm_medium=editorial-email&utm_source=authorletters&utm_campaign=protocols.

As the corresponding author, your ORCID iD is verified in the submission system and will appear in the published article. PLOS supports the use of ORCID, and we encourage all coauthors to register for an ORCID iD and use it as well. Please encourage your coauthors to verify their ORCID iD within the submission system before final acceptance, as unverified ORCID iDs will not appear in the published article. *Only*  the individual author can complete the verification step; PLOS staff *cannot*  verify ORCID iDs on behalf of authors.

We look forward to receiving your revised manuscript.

Kind regards,

Pengpeng Ye

Academic Editor

PLOS One

Journal Requirements:

When submitting your revision, we need you to address these additional requirements.

1. Please ensure that your manuscript meets PLOS ONE's style requirements, including those for file naming. The PLOS ONE style templates can be found at

https://journals.plos.org/plosone/s/file?id=wjVg/PLOSOne_formatting_sample_main_body.pdf and

https://journals.plos.org/plosone/s/file?id=ba62/PLOSOne_formatting_sample_title_authors_affiliations.pdf

2. Your ethics statement should only appear in the Methods section of your manuscript. If your ethics statement is written in any section besides the Methods, please move it to the Methods section and delete it from any other section. Please ensure that your ethics statement is included in your manuscript, as the ethics statement entered into the online submission form will not be published alongside your manuscript.

3. We note that Figure 2 in your submission contain map/satellite image which may be copyrighted. All PLOS content is published under the Creative Commons Attribution License (CC BY 4.0), which means that the manuscript, images, and Supporting Information files will be freely available online, and any third party is permitted to access, download, copy, distribute, and use these materials in any way, even commercially, with proper attribution. For these reasons, we cannot publish previously copyrighted maps or satellite images created using proprietary data, such as Google software (Google Maps, Street View, and Earth). For more information, see our copyright guidelines: http://journals.plos.org/plosone/s/licenses-and-copyright.

We require you to either (1) present written permission from the copyright holder to publish these figures specifically under the CC BY 4.0 license, or (2) remove the figures from your submission:

a. You may seek permission from the original copyright holder of Figure 2 to publish the content specifically under the CC BY 4.0 license.

We recommend that you contact the original copyright holder with the Content Permission Form (http://journals.plos.org/plosone/s/file?id=7c09/content-permission-form.pdf) and the following text:

“I request permission for the open-access journal PLOS ONE to publish XXX under the Creative Commons Attribution License (CCAL) CC BY 4.0 (http://creativecommons.org/licenses/by/4.0/). Please be aware that this license allows unrestricted use and distribution, even commercially, by third parties. Please reply and provide explicit written permission to publish XXX under a CC BY license and complete the attached form.”

Please upload the completed Content Permission Form or other proof of granted permissions as an "Other" file with your submission.

In the figure caption of the copyrighted figure, please include the following text: “Reprinted from [ref] under a CC BY license, with permission from [name of publisher], original copyright [original copyright year].”

b. If you are unable to obtain permission from the original copyright holder to publish these figures under the CC BY 4.0 license or if the copyright holder’s requirements are incompatible with the CC BY 4.0 license, please either i) remove the figure or ii) supply a replacement figure that complies with the CC BY 4.0 license. Please check copyright information on all replacement figures and update the figure caption with source information. If applicable, please specify in the figure caption text when a figure is similar but not identical to the original image and is therefore for illustrative purposes only.

The following resources for replacing copyrighted map figures may be helpful:

USGS National Map Viewer (public domain): http://viewer.nationalmap.gov/viewer/

The Gateway to Astronaut Photography of Earth (public domain): http://eol.jsc.nasa.gov/sseop/clickmap/

Maps at the CIA (public domain): https://www.cia.gov/library/publications/the-world-factbook/index.html and https://www.cia.gov/library/publications/cia-maps-publications/index.html

NASA Earth Observatory (public domain): http://earthobservatory.nasa.gov/

Landsat: http://landsat.visibleearth.nasa.gov/

USGS EROS (Earth Resources Observatory and Science (EROS) Center) (public domain): http://eros.usgs.gov/#

Natural Earth (public domain): http://www.naturalearthdata.com/

4. We notice that your supplementary table are uploaded with the file type 'Table'. Please amend the file type to 'Supporting Information'. Please ensure that each Supporting Information file has a legend listed in the manuscript after the references list.

5. Please include your tables as part of your main manuscript and remove the individual files. Please note that supplementary tables (should remain/ be uploaded) as separate "supporting information" files"

If the reviewer comments include a recommendation to cite specific previously published works, please review and evaluate these publications to determine whether they are relevant and should be cited. There is no requirement to cite these works unless the editor has indicated otherwise.

Please review your reference list to ensure that it is complete and correct. If you have cited papers that have been retracted, please include the rationale for doing so in the manuscript text, or remove these references and replace them with relevant current references. Any changes to the reference list should be mentioned in the rebuttal letter that accompanies your revised manuscript. If you need to cite a retracted article, indicate the article’s retracted status in the References list and also include a citation and full reference for the retraction notice

[Note: HTML markup is below. Please do not edit.]

Reviewers' comments:

Reviewer's Responses to Questions

**Comments to the Author**

1. Is the manuscript technically sound, and do the data support the conclusions?

Reviewer #1: Yes

**********

2. Has the statistical analysis been performed appropriately and rigorously?

Reviewer #1: Yes

**********

3. Have the authors made all data underlying the findings in their manuscript fully available?

The PLOS Data policy

Reviewer #1: Yes

**********

4. Is the manuscript presented in an intelligible fashion and written in standard English?

Reviewer #1: Yes

**********

Reviewer #1: This study, titled "The global burden of female breast cancer caused by high fasting plasma glucose: A comprehensive analysis of the GBD database from 1990 to 2023," examines the global impact of high fasting plasma glucose (HFPG) as a risk factor for female breast cancer (BC). Using data from the Global Burden of Disease (GBD) database, the study analyzes trends in deaths, disability-adjusted life years (DALYs), age-standardized mortality rates (ASMR), and age-standardized DALY rates (ASDR) from 1990 to 2023. It also predicts future trends up to 2050 using the Bayesian age-period-cohort (BAPC) model. The study highlights the growing global burden of female breast cancer caused by HFPG, particularly in developing regions. It emphasizes the need for targeted interventions, improved healthcare access, and preventive measures to address this public health challenge.

I congratulate the authors for conducting this all-important research study using GBD data.

The objectives of the study are well-defined, relevant, and address a critical gap in understanding the global burden of female breast cancer caused by HFPG. While the focus on HFPG is justified, future research could expand the scope to include other risk factors (Obesity, physical inactivity, dietary habits, genetic predisposition) and molecular subtypes to provide a more comprehensive understanding of breast cancer etiology and prevention strategies.

The study has several strengths, including its comprehensive scope, use of reliable data, and focus on a significant public health issue. However, it could be improved by addressing its narrow focus, exploring molecular subtypes (e.g. HER2-Positive, triple negative), incorporating additional biomarkers (e.g. HbA1C for diabetes), and providing more detailed policy recommendations. Despite these limitations, the study provides valuable insights into the global burden of female breast cancer caused by HFPG and highlights the need for targeted prevention and control strategies.

While the study mentions that ethical approval was not required, it would be helpful to explicitly state that the data used was anonymized and publicly available in the ethics section.

**********

what does this mean?). If published, this will include your full peer review and any attached files.

If you choose “no”, your identity will remain anonymous but your review may still be made public.

**Do you want your identity to be public for this peer review?**  For information about this choice, including consent withdrawal, please see our Privacy Policy

Reviewer #1: **Yes:**  U S H GAMAGE

**********

[NOTE: If reviewer comments were submitted as an attachment file, they will be attached to this email and accessible via the submission site. Please log into your account, locate the manuscript record, and check for the action link "View Attachments". If this link does not appear, there are no attachment files.]

To ensure your figures meet our technical requirements, please review our figure guidelines: https://journals.plos.org/plosone/s/figures

You may also use PLOS’s free figure tool, NAAS, to help you prepare publication quality figures: https://journals.plos.org/plosone/s/figures#loc-tools-for-figure-preparation.

NAAS will assess whether your figures meet our technical requirements by comparing each figure against our figure specifications.

# Author comment: Author response to Decision Letter 1

**DOI:** 10.1371/journal.pone.0359268.r002

23 Jul 2026

Thank you very much for your thoughtful and constructive comments on our manuscript. We sincerely appreciate the time and effort you have devoted to reviewing our work. Your insightful suggestions have been invaluable in helping us improve the quality of this paper. Below, we provide a point-by-point response to your comments.

Reviewer’s Comment 1:“The objectives of the study are well-defined, relevant, and address a critical gap in understanding the global burden of female breast cancer caused by HFPG. While the focus on HFPG is justified, future research could expand the scope to include other risk factors (Obesity, physical inactivity, dietary habits, genetic predisposition) and molecular subtypes to provide a more comprehensive understanding of breast cancer etiology and prevention strategies.”

Response: Thank you for your recognition of the study’s relevance and for this excellent suggestion. We fully agree that expanding the scope to include other risk factors (such as obesity, physical inactivity, dietary habits, and genetic predisposition) and exploring associations with molecular subtypes (e.g., HER2-positive, triple-negative) would provide a more holistic understanding of breast cancer etiology. We have added a paragraph in the Discussion section (Section 4) acknowledging this limitation and explicitly recommending that future studies incorporate multi-risk-factor analyses and molecular subtype stratification when data become available.

The revised text now reads:

“Looking to the future, it is necessary to integrate additional metabolic risk factors—such as obesity, physical inactivity, and dietary patterns—alongside HFPG, and explore their interactions with breast cancer molecular subtypes. Such efforts will be crucial for developing personalized prevention strategies.”

Reviewer’s Comment 2: “The study has several strengths, including its comprehensive scope, use of reliable data, and focus on a significant public health issue. However, it could be improved by addressing its narrow focus, exploring molecular subtypes (e.g. HER2-Positive, triple negative), incorporating additional biomarkers (e.g. HbA1C for diabetes), and providing more detailed policy recommendations.”

Response: We greatly appreciate your balanced assessment. As noted above, we have now explicitly discussed this limitation and called for broader, multi-factorial approaches in future work. Regarding molecular subtypes and additional biomarkers (e.g., HbA1c): We completely agree that HbA1c is a more stable indicator of long-term glycemic control than fasting plasma glucose alone. Unfortunately, the GBD database currently does not provide stratified data by molecular subtype or HbA1c levels.

Reviewer’s Comment 3: “While the study mentions that ethical approval was not required, it would be helpful to explicitly state that the data used was anonymized and publicly available in the ethics section.”

Response:Thank you for this practical suggestion. We have revised the Ethics approval and consent to participate section (Section 2.2) to explicitly state that all data used in this study are fully anonymized and publicly available from the GBD database. The revised text now reads:

“The GBD 2023 project is an open-access database in which all data are de-identified and publicly available. The University of Washington’s Institutional Review Board reviewed and approved an informed consent waiver (https://www.Healthdata.org/research-analysis/gbd). No additional ethical approval was required for this secondary analysis of anonymized, publicly available data.”

We believe these revisions adequately address all of your concerns. Once again, we thank you for your thorough review and valuable feedback, which have significantly strengthened our manuscript. We look forward to your further consideration.

Attachment Submitted filename: response to the reviewer.docx

# Peer review: Decision Letter 1

**DOI:** 10.1371/journal.pone.0359268.r003

**Authors:** Pengpeng Ye

25 Aug 2026

Dear Dr. Song,

Thank you for submitting your manuscript to PLOS One. After careful consideration, we feel that it has merit but does not fully meet PLOS One’s publication criteria as it currently stands. Therefore, we invite you to submit a revised version of the manuscript that addresses the points raised during the review process.

Please submit your revised manuscript by Oct 09 2026 11:59PM. If you will need more time than this to complete your revisions, please reply to this message or contact the journal office at plosone@plos.org. When you're ready to submit your revision, log on to https://www.editorialmanager.com/pone/ and select the 'Submissions Needing Revision' folder to locate your manuscript file.

- A letter that responds to each point raised by the academic editor and reviewer(s). You should upload this letter as a separate file labeled 'Response to Reviewers'.
- A marked-up copy of your manuscript that highlights changes made to the original version. You should upload this as a separate file labeled 'Revised Manuscript with Track Changes'.
- An unmarked version of your revised paper without tracked changes. You should upload this as a separate file labeled 'Manuscript'.

If you would like to make changes to your financial disclosure, please include your updated statement in your cover letter. Guidelines for resubmitting your figure files are available below the reviewer comments at the end of this letter.

If applicable, we recommend that you deposit your laboratory protocols in protocols.io to enhance the reproducibility of your results. Protocols.io assigns your protocol its own identifier (DOI) so that it can be cited independently in the future. For instructions see: https://journals.plos.org/plosone/s/submission-guidelines#loc-laboratory-protocols. Additionally, PLOS One offers an option for publishing peer-reviewed Lab Protocol articles, which describe protocols hosted on protocols.io. Read more information on sharing protocols at https://plos.org/protocols?utm_medium=editorial-email&utm_source=authorletters&utm_campaign=protocols.

As the corresponding author, your ORCID iD is verified in the submission system and will appear in the published article. PLOS supports the use of ORCID, and we encourage all coauthors to register for an ORCID iD and use it as well. Please encourage your coauthors to verify their ORCID iD within the submission system before final acceptance, as unverified ORCID iDs will not appear in the published article. *Only*  the individual author can complete the verification step; PLOS staff *cannot*  verify ORCID iDs on behalf of authors.

We look forward to receiving your revised manuscript.

Kind regards,

Pengpeng Ye

Academic Editor

PLOS One

Journal Requirements:

If the reviewer comments include a recommendation to cite specific previously published works, please review and evaluate these publications to determine whether they are relevant and should be cited. There is no requirement to cite these works unless the editor has indicated otherwise.

[Note: HTML markup is below. Please do not edit.]

Reviewers' comments:

Reviewer's Responses to Questions

**Comments to the Author**

Reviewer #1: All comments have been addressed

Reviewer #2: (No Response)

**********

2. Is the manuscript technically sound, and do the data support the conclusions?

Reviewer #1: Yes

Reviewer #2: No

**********

3. Has the statistical analysis been performed appropriately and rigorously?

Reviewer #1: Yes

Reviewer #2: No

**********

4. Have the authors made all data underlying the findings in their manuscript fully available?

The PLOS Data policy

Reviewer #1: Yes

Reviewer #2: Yes

**********

5. Is the manuscript presented in an intelligible fashion and written in standard English?

Reviewer #1: Yes

Reviewer #2: (No Response)

**********

Reviewer #1: The authors expressed sincere appreciation for the reviewers' feedback and provided a detailed, respectful, and constructive point-by-point response. This demonstrates professionalism and a willingness to improve the manuscript. Thank you.

a. Expanding Scope to Other Risk Factors and Molecular Subtypes

• The authors agreed with the suggestion, acknowledged the limitation, and added a paragraph in the Discussion section explicitly recommending future studies to incorporate multi-risk-factor analyses and molecular subtype stratification. They provided the revised text, showing transparency and a clear plan for future research.

b. Incorporating Additional Biomarkers (e.g., HbA1c) and Policy Recommendations

• The authors agreed that HbA1c is a more stable indicator than fasting plasma glucose but explained that the GBD database does not provide data stratified by HbA1c or molecular subtype. They acknowledged this as a limitation in the revised manuscript and called for broader, multi-factorial approaches in future work. This is a reasonable and transparent response given data constraints.

c. Clarifying Ethics and Data Anonymization

• The authors revised the Ethics section to clearly state that all data are anonymized and publicly available, and cited the relevant institutional review board approval and data source. They provided the revised text in their response.

3. Overall Quality of Revisions

• The authors made substantive changes to the manuscript in response to each major reviewer comment, including:

o Adding new discussion content to address limitations and future research directions.

o Clarifying ethical considerations and data sources.

o Transparently explaining any limitations due to data availability.

•The responses are specific, actionable, and directly address the reviewers' concerns, strengthening the manuscript.

Conclusion: The authors have addressed the reviewer’s comments thoroughly and professionally. They made appropriate revisions to the manuscript, acknowledged limitations where necessary, and provided clear explanations for any constraints. Their responses demonstrate a strong commitment to improving the quality and clarity of the research.

Reviewer #2: General Comments

This manuscript utilizes the Global Burden of Disease (GBD) 2023 dataset to describe the mortality and disability-adjusted life years (DALYs) of female breast cancer attributable to high fasting plasma glucose (HFPG). The authors also attempted to respond to the reviewers’ queries very well. Unfortunately, the current submission still suffers from significant methodological opacity, conceptual overreach, and a lack of statistical validation for its projections. The manuscript, in its present form, functions as a descriptive data update but fails to meet the rigorous standards for causal inference and predictive modeling despite attempts to upscale it after revision.

Major Compelling Points (Methodology and Design)

1. Conceptual and causal overreach

a) Serious concern: The manuscript repeatedly claims breast cancer is “caused by” HFPG. GBD comparative-risk assessments calculate attributable burden based on population exposure and pre-existing risk-outcome relationships; they do not establish causality within a new analysis. The authors must replace “caused by” with “attributable to” throughout the text, including the title.

b) Prevention recommendations: It is clear that the conclusion that these findings highlight an “urgency” for blood-glucose control as a breast cancer prevention strategy exceeds the evidence. This study is an ecological observation, not a clinical trial. It does not demonstrate that glycemic intervention reduces cancer incidence. I think the list to perfect here is framing as hypotheses for further investigation.

2. Methodological Scope

a) The authors treat HFPG interchangeably with diabetes. They must explicitly define the GBD exposure metric, specifically the Theoretical Minimum-Risk Exposure Level (TMREL). Without knowing the counterfactual (e.g., 4.8–5.4 mmol/L), the burden estimates lack biological context.

b) The analysis is restricted to "female" breast cancer without justification. While rare, male breast cancer is a recognized entity. The authors must state if this restriction is due to a lack of GBD risk-outcome pairs for males or a specific study design choice.

c) "Systematic Analysis”? This is a secondary analysis of a public database. Labeling it a “systematic analysis” is misleading as it does not follow PRISMA or similar systematic review protocols.

3. I have observed the authors attributed the rise in ASMR/ASDR solely to HFPG. However, they fail to account for the lag-time effect (the biological period between glucose exposure and oncogenesis). Furthermore, they must decompose the trend: how much is driven by changing HFPG prevalence versus changes in breast cancer survival, diagnostic intensity, or population aging?

Also, the authors conflate absolute numbers (48,307 deaths) with age-standardized rates. An increase in absolute burden is often a function of population growth and demographic aging rather than an increase in individual-level metabolic risk. These must be distinguished in the discussion.

4. The claim that SDI and BC-HFPG burden are linked is currently descriptive and prone to the Ecological Fallacy. Visual upward movement of two variables over 30 years does not establish an epidemiological link. A formal regression analysis or correlation coefficient (accounting for regional dependence) is required.

The term “developing regions” is not a formal GBD classification. The authors must use SDI quintiles (Low, Low-Middle, etc.) and provide quantitative proof that these areas are disproportionately affected compared to High-SDI regions.

5. Deficiencies in the BAPC forecasting model are grossly noted.

a) The Bayesian age-period-cohort (BAPC) model is a "black box" in this manuscript. The authors must provide out-of-sample validation (e.g., training the model on 1990–2015 data to see if it accurately predicts the observed 2016–2023 GBD estimates).

b) The projection begins in 2024, yet the current evaluation date is August 2026. The authors must explain why they are "predicting" years for which empirical data may already be accessible and clarify that 2024–2050 are model-based projections, not observed estimates.

c) The 2050 linear projection fails to account for the current global shift in metabolic management (e.g., the rapid uptake of GLP-1 receptor agonists). The authors must discuss how revolutionary changes in global glucose management might render their 26-year linear forecast obsolete.

Minor and Technical Issues

1. The Results state a trend analysis from 1992–2023, yet the reported values and Table 1 refer to 1990–2023. This inconsistency must be resolved to ensure the integrity of the AAPC estimates.

2. Average Annual Percent Changes (AAPC) are reported as 0.28% and 0.44%. The authors must provide 95% Confidence Intervals (CIs) in the text (not just tables) and state whether these changes are statistically distinguishable from zero.

3. Figures 2 and 3 must include uncertainty intervals to prevent an appearance of false precision in the long-term forecasts.

**********

what does this mean?). If published, this will include your full peer review and any attached files.

If you choose “no”, your identity will remain anonymous but your review may still be made public.

**Do you want your identity to be public for this peer review?**  For information about this choice, including consent withdrawal, please see our Privacy Policy

Reviewer #1: **Yes:**  UDAYA SAMANTHILAKA HATTOTUWA GAMAGE

Reviewer #2: **Yes:**  Kamoru A. Adedokun

**********

[NOTE: If reviewer comments were submitted as an attachment file, they will be attached to this email and accessible via the submission site. Please log into your account, locate the manuscript record, and check for the action link "View Attachments". If this link does not appear, there are no attachment files.]

To ensure your figures meet our technical requirements, please review our figure guidelines: https://journals.plos.org/plosone/s/figures

You may also use PLOS’s free figure tool, NAAS, to help you prepare publication quality figures: https://journals.plos.org/plosone/s/figures#loc-tools-for-figure-preparation.

NAAS will assess whether your figures meet our technical requirements by comparing each figure against our figure specifications.

Attachment Submitted filename: REVIEWER REPORT.pdf

# Author comment: Author response to Decision Letter 2

**DOI:** 10.1371/journal.pone.0359268.r004

4 Sep 2026

Response to Reviewers

Manuscript Title: The global burden of female breast cancer attributable to high fasting plasma glucose: A comprehensive analysis of the GBD database from 1990 to 2023

Dear Editor and Reviewers,

We thank you and the reviewers for the time and effort devoted to our manuscript. In this revised manuscript (page and line numbers are provided; line numbers restart on each page), we have carefully addressed every comment. All changes are highlighted in red in the revised manuscript, and point-by-point responses are provided below; page and line numbers in each response refer to the revised marked manuscript. Reviewer comments are shown in italics, followed by our responses.

Sincerely,

Huadi Shi, Zhi Song, Yaoyao Chen, Siyuan Liu, Yuling Song (on behalf of all authors)

Response to Reviewer #1

The authors expressed sincere appreciation for the reviewers' feedback and provided a detailed, respectful, and constructive point-by-point response... Conclusion: The authors have addressed the reviewer's comments thoroughly and professionally.

Response: We sincerely thank the reviewer for the positive evaluation of our previous revisions. In the current revision, we have further strengthened the manuscript in response to Reviewer #2’s comments, including a systematic revision of causal-attribution language (Title, p. 1, lines 1–2; throughout), explicit definitions of the exposure metric (Methods 2.1, p. 4, lines 10–25), formal statistical analyses of the SDI–burden association (Methods 2.3, p. 5, lines 15–28; Results 3.4, p. 10, lines 1–25), and an out-of-sample validation of the projection model (Methods 2.3, p. 5, line 29 to p. 6, line 14; Results 3.5, p. 11, line 25 to p. 12, line 5). The ethics statement has also been expanded (Methods 2.2, p. 4, line 26 to p. 5, line 2), the HbA1c limitation acknowledged (Discussion, Limitations, p. 15, lines 19–23), and future multi-risk-factor and molecular-subtype research recommended (p. 15, lines 23–28).

Response to Reviewer #2

General Response

Unfortunately, the current submission still suffers from significant methodological opacity, conceptual overreach, and a lack of statistical validation for its projections...

Response: We thank the reviewer for this rigorous and constructive critique. We fully accept the core point: a GBD-based attributable-burden analysis is a descriptive, ecological assessment and must not be framed as causal inference or validated prediction. In this revision we have: (1) replaced causal wording with attribution language throughout, including the title (p. 1, lines 1–2); (2) reframed prevention-related conclusions as hypotheses for future research (Abstract Conclusion, p. 2, lines 11–18; Conclusion, p. 16, lines 9–15); (3) explicitly defined the exposure metric, HFPG above the Theoretical Minimum-Risk Exposure Level (TMREL), and distinguished it from diabetes (Methods 2.1, p. 4, lines 10–20); (4) justified the restriction to female breast cancer and removed the “systematic analysis” label (p. 4, lines 21–25; Abstract Methods, p. 1, lines 7–15); (5) added discussion of the lag-time effect and demographic drivers of the absolute burden (Results 3.1, p. 6, line 21 to p. 7, line 7; Discussion, p. 14, lines 15–27); (6) performed formal correlation analyses of the SDI–burden association and replaced “developing regions” with SDI quintile terminology (Results 3.4, p. 10, lines 1–25); and (7) performed an out-of-sample validation of the BAPC model, added an explicit statement that 2024–2050 values are model-based projections, and discussed how emerging metabolic therapies may affect long-term forecasts (Methods 2.3, p. 5, line 29 to p. 6, line 14; Results 3.5, p. 11, line 25 to p. 12, line 5; Discussion, Limitations, p. 15, line 29 to p. 16, line 7).

Comment 1a. Conceptual and causal overreach — “caused by”

"The manuscript repeatedly claims breast cancer is 'caused by' HFPG... The authors must replace 'caused by' with 'attributable to' throughout the text, including the title."

Response: We fully agree. The GBD comparative risk assessment quantifies attributable burden using population exposure distributions and pooled relative risks from prospective studies; it does not establish causality within our own analysis. We have replaced "caused by" with "attributable to" throughout, including the title, which now reads: "The global burden of female breast cancer attributable to high fasting plasma glucose: A comprehensive analysis of the GBD database from 1990 to 2023" (p. 1, lines 1–2). All corresponding instances in the Abstract (p. 1, line 16 to p. 2, line 10), Results 3.3 (p. 9, lines 13–29), Discussion (p. 13, line 2), and Conclusion (p. 16, lines 9–15) have been revised accordingly.

Changes made: Title (p. 1, lines 1–2); Abstract (p. 1, line 16 to p. 2, line 10); Results 3.3 (p. 9, lines 13–29); Discussion (p. 13, line 2); Conclusion (p. 16, lines 9–15).

Comment 1b. Prevention recommendations exceed the evidence

"...the conclusion that these findings highlight an 'urgency' for blood-glucose control as a breast cancer prevention strategy exceeds the evidence... framing as hypotheses for further investigation."

Response: We agree completely. As an ecological, population-level analysis, our study cannot demonstrate that glycemic intervention reduces breast cancer incidence. We have removed the word "urgency" and rewritten the Conclusion (and the corresponding sentences in the Abstract) to frame glycemic management and metabolic risk-factor control as considerations within comprehensive prevention frameworks and as hypotheses for future research, explicitly stating that "as this is an ecological, population-level analysis, whether glucose-lowering interventions can reduce breast cancer incidence remains to be established in prospective interventional studies." Prevention-oriented recommendations in the Discussion (p. 14, line 28 to p. 15, line 14) have likewise been framed as suggestions for high-burden regions rather than claims of established benefit.

Changes made: Abstract Conclusion (p. 2, lines 11–18); Conclusion (p. 16, lines 9–15); Discussion recommendations (p. 14, line 28 to p. 15, line 14).

Comment 2a. HFPG must be explicitly defined, including TMREL; HFPG is not diabetes

"The authors treat HFPG interchangeably with diabetes. They must explicitly define the GBD exposure metric, specifically the Theoretical Minimum-Risk Exposure Level (TMREL)..."

Response: Thank you for this important comment. We have added an explicit definition in Methods 2.1 (p. 4, lines 10–20): HFPG is defined as fasting plasma glucose exceeding the Theoretical Minimum-Risk Exposure Level (TMREL), approximately 4.8–5.4 mmol/L (86.4–97.2 mg/dL) in the GBD 2023 risk factor analysis. We now state explicitly that (1) the burden estimates represent the fraction of female breast cancer burden attributable to HFPG under the TMREL counterfactual, as modeled by GBD using pooled relative risks from prospective cohort studies; and (2) HFPG is a continuous exposure above the TMREL and is not equivalent to a clinical diagnosis of diabetes mellitus, capturing the full spectrum of elevated fasting glucose including prediabetic ranges. In addition, where diabetes studies are cited in the Introduction, we added transitional wording (p. 3, lines 8–20) clarifying that they provide supportive evidence for a related but narrower exposure construct; a parallel caveat was added at the end of the mechanistic Discussion paragraph (p. 12, line 26 to p. 13, line 13) and in the Limitations (p. 15, lines 19–23) noting that our estimates are not directly comparable with studies of diabetes-attributable breast cancer burden.

Changes made: Methods 2.1, new definitional paragraph (p. 4, lines 10–20); Introduction transitional wording (p. 3, lines 8–20); Discussion mechanism caveat (p. 12, line 26 to p. 13, line 13); Limitations (p. 15, lines 19–23).

Comment 2b. Justify the restriction to female breast cancer

"The analysis is restricted to 'female' breast cancer without justification..."

Response: We have clarified this in Methods 2.1 (p. 4, lines 21–25): the analysis was restricted to female breast cancer because the GBD comparative risk assessment models HFPG as a risk factor for female breast cancer only; no risk–outcome pair between HFPG and male breast cancer has been established in the GBD framework, owing to the rarity of male breast cancer and the limited evidence base for quantitative risk estimation. This is a data-availability constraint rather than a design choice, and we now state this explicitly.

Changes made: Methods 2.1 (p. 4, lines 21–25).

Comment 2c. "‘Systematic Analysis’" is misleading

"‘Systematic Analysis’? This is a secondary analysis of a public database..."

Response: We agree and have removed this label. The Abstract Methods now describes the study as "a comprehensive descriptive analysis of the burden of female BC-HFPG based on the GBD 2023 database" (p. 1, lines 7–15). (Reference titles containing "a systematic analysis," which refer to the GBD collaborators’ own papers, are retained as published.)

Changes made: Abstract Methods (p. 1, lines 7–15); Methods 2.3 (p. 5, lines 15–28).

Comment 3. Lag-time effect, trend decomposition, and absolute numbers vs. age-standardized rates

"The authors attributed the rise in ASMR/ASDR solely to HFPG... lag-time effect... decompose the trend... conflate absolute numbers (48,307 deaths) with age-standardized rates."

Response: We thank the reviewer for this insightful comment and have addressed it in three ways:

(1) Absolute vs. age-standardized burden. Results 3.1 now explicitly reports the divergence (p. 6, line 21 to p. 7, line 7): the absolute number of BC-HFPG deaths increased by 150.0% (from 19,328 in 1990 to 48,307 in 2023), whereas the ASMR rose by only 8.7% (from 0.92 to 1.00 per 100,000). We state that this divergence indicates the growth in absolute burden is largely driven by population growth and demographic aging, whereas the modest rise in age-standardized rates reflects an increase in individual-level metabolic risk beyond demographic change.

(2) Lag-time effect. A new Discussion paragraph (p. 14, lines 15–27) acknowledges that oncogenesis following metabolic exposure involves a substantial lag period; the burden observed in any given year partly reflects HFPG exposure from years to decades earlier, so recent changes in diabetes screening and management will only become visible in BC-HFPG trends after a delay.

(3) Trend decomposition. The same paragraph (p. 14, lines 15–27) acknowledges that aggregate GBD estimates do not permit formal decomposition of the trends into changing HFPG prevalence, breast cancer survival, diagnostic intensity, and population aging; the divergent trajectories of counts versus rates suggest demographic change is the dominant driver of the absolute burden, and we call for future studies employing formal decomposition methods (e.g., age-period-cohort decomposition).

Changes made: Results 3.1 (p. 6, line 21 to p. 7, line 7); Discussion, new paragraph on temporal-trend interpretation (p. 14, lines 15–27).

Comment 4. Ecological fallacy of the SDI association; "developing regions" is not a formal GBD classification

"The claim that SDI and BC-HFPG burden are linked is currently descriptive and prone to the Ecological Fallacy... A formal regression analysis or correlation coefficient (accounting for regional dependence) is required... use SDI quintiles..."

Response: We agree, and have made the following revisions:

(1) Formal correlation analyses (Methods 2.3, p. 5, lines 15–28; Results 3.4, p. 10, lines 1–25). Pearson and Spearman correlation coefficients with 95% CIs from 1,000 bootstrap resamples were calculated between the 2023 SDI and ASMR/ASDR across all 204 countries and territories. SDI showed a weak positive correlation with ASMR (Pearson r = 0.13, 95% CI: 0.02–0.24, P = 0.073; Spearman ρ = 0.20, 95% CI: 0.06–0.32, P = 0.004) and no significant correlation with ASDR (Pearson r = 0.03, 95% CI: −0.08 to 0.15; Spearman ρ = 0.09, 95% CI: −0.04 to 0.24, P = 0.196). To account for regional dependence, a sensitivity analysis at the level of the 21 GBD regions showed that the correlations attenuated and reversed direction (ASMR: r = −0.37, P = 0.101; ASDR: r = −0.46, P = 0.036) (new Fig. 4, caption at p. 11, lines 13–17). LOESS curves revealed a non-linear, inverted-U-shaped relationship peaking at an SDI of approximately 0.75 (new Fig. 3, caption at p. 11, lines 4–12). These coefficients are interpreted strictly as ecological associations.

(2) SDI quintile terminology. "Developing regions" and similar informal terms have been replaced with GBD’s formal SDI quintile classification throughout (Abstract Conclusion, p. 2, lines 11–18; Discussion, p. 13, line 18 to p. 14, line 14).

(3) Quantitative evidence across SDI quintiles (Results 3.4, p. 10, lines 17–25). In 2023, both ASMR and ASDR were highest in the low-middle SDI quintile (ASMR 1.22, 95% UI: 0.70–2.12; ASDR 37.7, 95% UI: 21.7–67.5 per 100,000), with statistically significant inter-quintile differences (Kruskal–Wallis H = 29.9 for ASMR and 25.7 for ASDR, both P < 0.001). The low-SDI quintile had a 1.18-fold higher ASDR than the high-SDI quintile (32.6 vs. 27.6 per 100,000), whereas ASMR was comparable (0.98 vs. 0.98 per 100,000). The revised text states that the burden is non-linearly distributed across the development spectrum, peaking in low-middle SDI countries, rather than making a blanket claim about "developing regions."

Changes made: Methods 2.3 (p. 5, lines 15–28); Results 3.4 (p. 10, lines 1–25) with new Figs. 2–4 (Fig. 2 caption p. 10, line 26 to p. 11, line 3; Fig. 3 caption p. 11, lines 4–12; Fig. 4 caption p. 11, lines 13–17); Abstract Conclusion (p. 2, lines 11–18); Discussion (p. 13, line 18 to p. 14, line 14).

Comment 5a. Out-of-sample validation of the BAPC model

"The Bayesian age-period-cohort (BAPC) model is a 'black box'... provide out-of-sample validation (e.g., training the model on 1990–2015 data to see if it accurately predicts the observed 2016–2023 GBD estimates)."

Response: We agree that model validation is essential. We performed a temporal out-of-sample validation: the BAPC model was trained on 1990–2015 data and used to retroactively predict ASMR and ASDR for the held-out 2016–2023 period, which were compared with observed GBD 2023 estimates. The procedure is described in Methods 2.3 (p. 5, line 29 to p. 6, line 14), and the results in Results 3.5 (p. 11, line 25 to p. 12, line 5) with the observed-versus-predicted comparison shown in new Fig. 6 (caption at p. 12, lines 14–18). Predictive accuracy was high: MAPE = 1.88% for ASMR (RMSE = 0.051 per 100,000) and 2.06% for ASDR (RMSE = 1.485 per 100,000), and all eight held-out observations fell within the corresponding 95% prediction intervals (coverage = 100%). Notably, prediction errors increased monotonically with the forecast horizon, from below 1.3% for one- to five-year-ahead predictions to approximately 6% by year eight, and the model systematically under-predicted the accelerated burden observed in 2021–2023. These findings underpin an explicit statement in Results 3.5 that longer-range projections, including those to 2050, carry substantial uncertainty and should be interpreted with caution.

Changes made: Methods 2.3 (p. 5, line 29 to p. 6, line 14); Results 3.5 (p. 11, line 25 to p. 12, line 5); new Fig. 6 (caption at p. 12, lines 14–18).

Comment 5b. Projection starting in 2024

"The projection begins in 2024, yet the current evaluation date is August 2026..."

Response: We clarify that official GBD estimates are currently published through 2023 only (GBD 2023 round); no empirical GBD estimates exist for 2024 or later, so our projection period does not overlap with any published empirical estimates. All values presented for 2024–2050 are model-based projections generated by the BAPC model and do not represent observed estimates; this is now stated explicitly in Methods 2.3 (p. 6, lines 11–14) and in the Fig. 5 legend (p. 12, lines 6–13).

Changes made: Methods 2.3 (p. 6, lines 11–14); Results 3.5 (p. 11, line 18 to p. 12, line 5); Fig. 5 legend (p. 12, lines 6–13).

Comment 5c. Long-term projections do not account for changing metabolic management (GLP-1 receptor agonists)

"The 2050 linear projection fails to account for the current global shift in metabolic management (e.g., the rapid uptake of GLP-1 receptor agonists)..."

Response: We agree. The Limitations now note that our projections to 2050 assume historical exposure and mortality trends continue unchanged, whereas the rapid global uptake of novel metabolic therapies—most notably GLP-1 receptor agonists—together with expanding diabetes screening and management programmes could substantially alter future HFPG exposure trajectories. If these interventions translate into sustained population-level reductions in fasting glucose, the projected rise in BC-HFPG burden may be overestimated; our forecasts should be interpreted as a reference scenario conditional on the continuation of current trends rather than a prediction of an inevitable future (p. 15, line 29 to p. 16, line 7).

Changes made: Discussion, Limitations (p. 15, line 29 to p. 16, line 7).

Minor Comment 1. Inconsistency between 1992–2023 and 1990–2023

"The Results state a trend analysis from 1992–2023, yet the reported values and Table 1 refer to 1990–2023..."

Response: We apologize for this typographical inconsistency. All trend descriptions now uniformly refer to 1990–2023, consistent with Table 1 (p. 7, line 8 to p. 8) and the AAPC estimates (Abstract Results, p. 1, line 16 to p. 2, line 10; Results 3.1, p. 6, line 21 to p. 7, line 7).

Changes made: Abstract Results (p. 1, line 16 to p. 2, line 10); Results 3.1 (p. 6, line 21 to p. 7, line 7).

Minor Comment 2. AAPC confidence intervals in the text

"Average Annual Percent Changes (AAPC) are reported as 0.28% and 0.44%. The authors must provide 95% Confidence Intervals (CIs) in the text..."

Response: We now report the AAPCs with 95% CIs in both the Abstract and the Results text: the ASMR AAPC was 0.28% (95% CI: 0.18–0.38%) and the ASDR AAPC was 0.44% (95% CI: 0.37–0.51%). Both confidence intervals exclude zero, indicating statistically significant increasing trends (Abstract Results, p. 1, line 16 to p. 2, line 10; Results 3.1, p. 6, lines 27–29 and p. 7, lines 1–2).

Changes made: Abstract Results (p. 1, line 16 to p. 2, line 10); Results 3.1 (p. 6, lines 27–29 to p. 7, lines 1–2).

Minor Comment 3. Uncertainty intervals in Figures 2 and 3

"Figures 2 and 3 must include uncertainty intervals to prevent an appearance of false precision in the long-term forecasts."

Response: We have revised all forecast-related figures to display their underlying uncertainty. The forecast figure (Fig. 5, p. 12, lines 6–13) now shows 95% uncertainty intervals for the observed period (1990–2023) and 95% prediction intervals for the projected period (2024–2050), with the legend specifying the meaning of the lines and shaded bands and stating explicitly that 2024–2050 values are model-based projections rather than observed estimates. The regional frontier figure (Fig. 2, p. 10, line 26 to p. 11, line 3) now displays 95% uncertainty intervals of the GBD estimates as error bars at 5-year intervals, and the SDI–burden association figure (Fig. 3, p. 11, lines 4–12) shows LOESS fits with 95% confidence bands. In addition, the out-of-sample validation (Fig. 6, p. 12, lines 14–18) provides direct empirical evidence of forecast uncertainty: prediction errors grow from <1.3% at short horizons to ≈6% at eight years, which we cite when urging caution about long-range extrapolations (p. 11, line 25 to p. 12, line 5).

Changes made: Fig. 5 and legend (p. 12, lines 6–13); Fig. 2 and legend (p. 10, line 26 to p. 11, line 3); Fig. 3 and legend (p. 11, lines 4–12); Fig. 6 and legend (p. 12, lines 14–18); Results 3.5 (p. 11, line 25 to p. 12, line 5).

Closing

We hope that these revisions have fully addressed the reviewers’ concerns. We are grateful for the opportunity to improve our manuscript and welcome any further suggestions.

Sincerely,

Huadi Shi, Zhi Song, Yaoyao Chen, Siyuan Liu, Yuling Song

On behalf of all authors

Attachment Submitted filename: Response_to_Reviewers_9.5.docx

# Peer review: Decision Letter 2

**DOI:** 10.1371/journal.pone.0359268.r005

**Authors:** Pengpeng Ye

13 Sep 2026

<p>The global burden of female breast cancer attributable to high fasting plasma glucose: A comprehensive analysis of the GBD database from 1990 to 2023

PONE-D-25-58557R2

Dear Dr. Song,

We’re pleased to inform you that your manuscript has been judged scientifically suitable for publication and will be formally accepted for publication once it meets all outstanding technical requirements.

Within one week, you’ll receive an e-mail detailing the required amendments. When these have been addressed, you’ll receive a formal acceptance letter and your manuscript will be scheduled for publication.

An invoice will be generated when your article is formally accepted. Please note, if your institution has a publishing partnership with PLOS and your article meets the relevant criteria, all or part of your publication costs will be covered. Please make sure your user information is up-to-date by logging into Editorial Manager at Editorial Manager® and clicking the ‘Update My Information' link at the top of the page. For questions related to billing, please contact billing support.

If your institution or institutions have a press office, please notify them about your upcoming paper to help maximize its impact. If they’ll be preparing press materials, please inform our press team as soon as possible -- no later than 48 hours after receiving the formal acceptance. Your manuscript will remain under strict press embargo until 2 pm Eastern Time on the date of publication. For more information, please contact onepress@plos.org.

Kind regards,

Pengpeng Ye

Academic Editor

PLOS One

Additional Editor Comments (optional):

Reviewers' comments:

Reviewer's Responses to Questions

**Comments to the Author**

Reviewer #2: All comments have been addressed

**********

2. Is the manuscript technically sound, and do the data support the conclusions?

Reviewer #2: Yes

**********

3. Has the statistical analysis been performed appropriately and rigorously?

Reviewer #2: Yes

**********

4. Have the authors made all data underlying the findings in their manuscript fully available?

The PLOS Data policy

Reviewer #2: Yes

**********

5. Is the manuscript presented in an intelligible fashion and written in standard English?

Reviewer #2: Yes

**********

Reviewer #2: The authors have revised the manuscript satisfactorily. Therefore, I have not other comments against the manuscript.

**********

what does this mean?). If published, this will include your full peer review and any attached files.

If you choose “no”, your identity will remain anonymous but your review may still be made public.

**Do you want your identity to be public for this peer review?**  For information about this choice, including consent withdrawal, please see our Privacy Policy

Reviewer #2: **Yes:**  Kamoru A. Adedokun

**********

# Editor report: Acceptance letter

**DOI:** 10.1371/journal.pone.0359268.r006

**Authors:** Pengpeng Ye

PONE-D-25-58557R2

PLOS One

Dear Dr. Song,

I'm pleased to inform you that your manuscript has been deemed suitable for publication in PLOS One. Congratulations! Your manuscript is now being handed over to our production team.

At this stage, our production department will prepare your paper for publication. This includes ensuring the following:

* All references, tables, and figures are properly cited

* All relevant supporting information is included in the manuscript submission,

* There are no issues that prevent the paper from being properly typeset

You will receive further instructions from the production team, including instructions on how to review your proof when it is ready. Please keep in mind that we are working through a large volume of accepted articles, so please give us a few days to review your paper and let you know the next and final steps.

Lastly, if your institution or institutions have a press office, please let them know about your upcoming paper now to help maximize its impact. If they'll be preparing press materials, please inform our press team within the next 48 hours. Your manuscript will remain under strict press embargo until 2 pm Eastern Time on the date of publication. For more information, please contact onepress@plos.org.

You will receive an invoice from PLOS for your publication fee after your manuscript has reached the completed accept phase. If you receive an email requesting payment before acceptance or for any other service, this may be a phishing scheme. Learn how to identify phishing emails and protect your accounts at https://explore.plos.org/phishing.

If we can help with anything else, please email us at customercare@plos.org.

Thank you for submitting your work to PLOS One and supporting open access.

Kind regards,

PLOS One Editorial Office Staff

on behalf of

Dr. Pengpeng Ye

Academic Editor

PLOS One
